# Spatiotemporal dynamics and risk factors for human Leptospirosis in Brazil

**DOI:** 10.1038/s41598-018-33381-3

**Published:** 2018-10-11

**Authors:** Oswaldo Santos Baquero, Gustavo Machado

**Affiliations:** 1 Department of Preventive Veterinary Medicine and Animal Health, School of Veterinary Medicine and Animal Science, University of São Paulo, Av. Prof. Orlando Marques de Paiva, 87, Cidade Universitária, São Paulo, SP CEP: 05508-270 Brazil; 2 Department of Population Health and Pathobiology, College of Veterinary Medicine, North Carolina State University, 1060 William Moore Drive, Raleigh, NC 27607 USA

**Keywords:** Risk factors, Bacterial infection

## Abstract

Leptospirosis is an emerging neglected tropical disease with a worldwide significant global health burden. Between 2000 and 2016, there were 63,302 cases of human leptospirosis and 6,064 deaths reported in Brazil. We modeled the spatiotemporal risk dynamics of human leptospirosis morbidity and lethality, and attributed an easily interpretable risk-based priority index (PI) for all Brazilian federative units to suggest improvements to the national surveillance system. We also developed a conceptual framework of causality and estimated the effects of environmental and socioeconomic determinants of morbidity and lethality. Spatiotemporal risk patterns of morbidity and lethality differed. For morbidity, the pattern was mainly spatial, whereas lethality was mainly explained by the spatiotemporal interaction. The hypothesized causal model explained a relevant fraction of the heterogeneity in the spatial and spatiotemporal interaction patterns. The increase in soil moisture, precipitation, poverty, and the decrease in the proportion of urban households, acted as risk factors. The increase in the proportion of households in which waste is directly collected and in temperature were preventive factors. The structured temporal trend was increasing for morbidity and decreasing for lethality. In terms of morbidity, it was clear that the prioritization should be focused in a couple of states, mainly Acre. In terms of lethality, the allocation of resources need not be as asymmetric, but there was nonetheless a prioritization order. The proposed approach can be used to characterize spatiotemporal dynamics of other diseases and to inform decision makers.

## Introduction

Leptospirosis is a global worldwide emerging zoonotic disease, caused by a spirochete bacterium of the genus *Leptospira*^1^. Leptospirosis is one of the leading global causes of morbidity and mortality, and it is estimated that the greatest burdens are in resource-poor regions and in areas where surveillance is not routinely performed^2^. Each year, there are an estimated 1.0 million cases of Leptospirosis and 58,900 deaths caused by the disease worldwide. In Andean and South Latin America, the annual estimated morbidity rates ranged from 1.43 to 39.8 per 100,000 population^2^; notably, while Brazil had 40.2% of the reported cases, this country has almost half of the total population of Latin America. Brazil was followed in proportion of reported cases by Peru (23.6%), Colombia (8.8%), and Ecuador (7.2%)^3^.

Leptospirosis is often associated with fever, headache, myalgia, and weakness^4^, and may be confused with other disease entities such as influenza and dengue fever^5^. It has been estimated that 10% to 15% of leptospirosis cases show severe clinical signs, a form of the infection known as Weil’s syndrome, and such cases are more likely to be correctly diagnosed^6^. The consequences of severe clinical signs are often associated with lethality^6^. Brazil follows the World Health Organization (WHO) guides to identify and confirm cases of leptospirosis. Leptospirosis is a disease requiring compulsory notification in Brazil, and confirmation of leptospirosis cases depends on certain laboratory (enzyme-linked immunosorbent assay [ELISA] test, microagglutination reaction, and blood or urine cultures) and clinical-epidemiological criteria^7^.

Leptospires have a preference for warm-blooded animals, mainly mammals^8^. The organism is highly adaptable to environmental conditions and can survive for long periods in water and wet soil^9,10^. Disease transmission is favored by climatic variables such as heavy rain, floods, and high temperatures^11,12^. Socioeconomic variables including occupation and poverty, among others, might also have implications for disease transmission because they can increase exposure to environments with rodent infestation, flooded soils, and other risk factors^13^.

Although many studies have described the spatiotemporal variability of human leptospirosis occurrences, and multiple environmental and socioeconomic factors have been associated with the disease, there is a deficiency in studies characterizing the spatiotemporal dynamics of the risk of human leptospirosis in an entire country, aimed at identifying which regions should be prioritized and to what extent. Moreover, there is a need to synthesize previous findings regarding risk factors, in a conceptual framework of causality that can be statistically tested.

Analytical epidemiological studies at the individual level might be too expensive to be implemented in an entire country, especially in large countries such as Brazil that still faces major development challenges. Ecological studies are an alternative because they use aggregates as units of analysis and aggregated data are widely available for all the main administrative areas of many countries. However, if reporting bias vary across aggregates, ecological results will be partially explained by this bias. With conceptual frameworks of causality one can identify potential risk factors that in ecological studies become relevant predictors. This predictors are useful for surveillance and decision making but it should be remembered that predictive contributions estimated in ecological studies provide no statistical support for individual level hypothesis.

The objectives of the present study were to (i) model the spatiotemporal dynamics of the risk of human leptospirosis morbidity and lethality, across all Brazilian states and the Federal District, from 2000 to 2016; (ii) fit statistical spatiotemporal models to estimate the effect of environmental and socioeconomic determinants conceptualized in a framework of causality; and (iii) suggest, based on risk estimates, the priority that should be given to each Brazilian state.

## Methods

This section is divided into six subsections. The first presents the data and their sources, while the second and third describe the conceptual framework of our hypothesis, and the statistical models that represented the conceptual framework. The fourth describes the model’s priors, while the last two present the model diagnostic procedures and software utilized, respectively.

### Data source and collection

The count number of cases and deaths of human leptospirosis, and the number of dengue cases, were obtained from the National Information System of Health of the Ministry of Health (Sistema de Informação de Agravos de Notificação [SINAN] do Ministério da Saúde [MS])^14^. To test the conceptual framework of causality described below, we collected secondary environmental and socioeconomic covariates. The environmental covariates, obtained from TerraClimate^15^, consisted of average values of descriptive statistics (minimum, mean, maximum, range, standard, deviation, sum), calculated over a grid of approximately 4 km^2^. The socioeconomic covariates were obtained from the Brazilian Institute of Geography and Statistics (IBGE)^16^ and the Institute of Applied Economic Research (IPEA)^17^. All variables were centered by subtracting their respective mean and scaled by dividing the centered values to the standard deviation. Table 1 illustrates the variables and data sources.

**Table 1:** Data description and sources, 2000–2016.

Variable	Description (unit)	Source
Cases	Annual number of human leptospirosis cases	SINAN - portalarquivos.saude.gov.br/…/Leptospirose-casos-05_2017.pdf
Deaths	Annual number of human leptospirosis deaths	SINAN - portalarquivos.saude.gov.br/…/Leptospirose-obitos-05_2017.pdf
Precipitation	Annual average of *, from monthly raster of spatial resolution of 1/24. ~ 4 km^2^ (mm)	TerraClimate - Web https://climate.northwestknowledge.net/TERRACLIMATE/index_directDownloads.php
Temperature	Annual average of *, from monthly raster of spatial resolution of 1/24. ~ 4 km^2^ (°C)	TerraClimate - Web https://climate.northwestknowledge.net/TERRACLIMATE/index_directDownloads.php
Soil moisture	Annual average of *, from monthly raster of spatial resolution of 1/24. ~ 4 km^2^ (mm)	TerraClimate - Web https://climate.northwestknowledge.net/TERRACLIMATE/index_directDownloads.php
HH poverty	Proportion of households (HH) in poverty (%)	IPEA - www.ipeadata.gov.br/
HH urban	Proportion of urban households (%)	IBGE - https://sidra.ibge.gov.br
Illiterates	Proportion of illiterate residents aged 15 years or older (%)	IPEA - www.ipeadata.gov.br/
HH collected waste	Proportion of households in which waste is directly collected (%)	IBGE - https://sidra.ibge.gov.br
Dengue cases	Incidence of dengue cases	SINAN - portalms.saude.gov.br/images/pdf/…/Dengue-classica-ate-2016.pdf
Brazil	Shapefile with Brazilian states	IBGE - https://mapas.ibge.gov.br/bases-e-referenciais/bases-cartograficas/malhas-digitais.html

^*^Minimum, mean, maximum, range, standard deviation, and sum.

### Conceptual framework of causality

**Figure 1:**
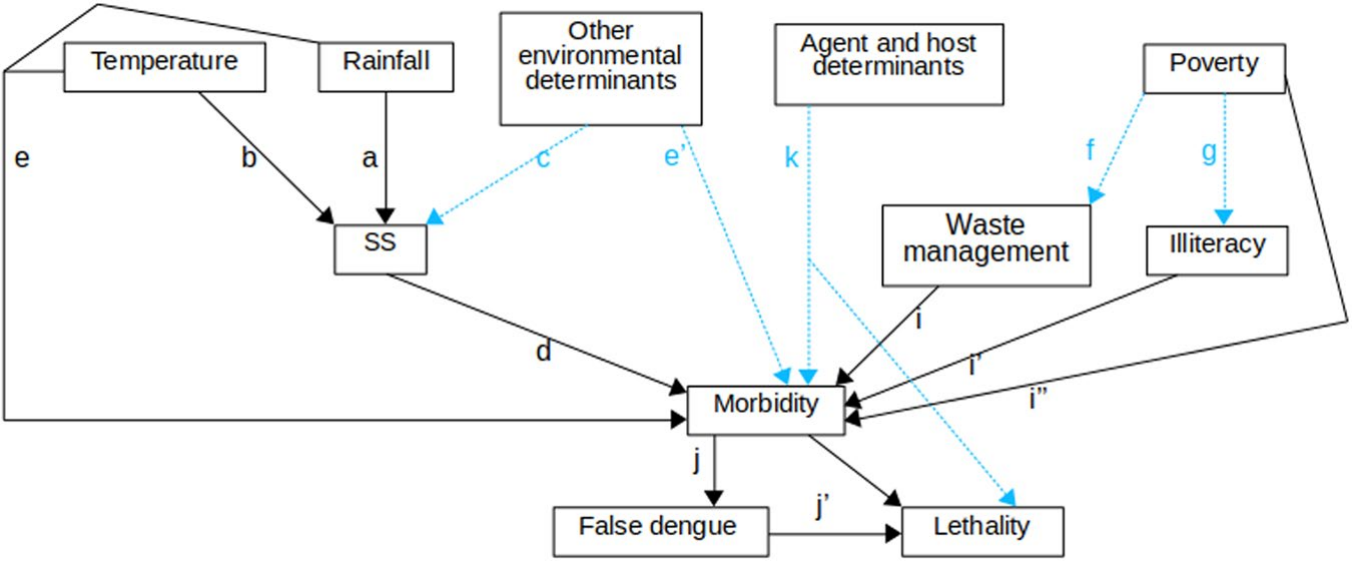
Conceptual framework of causality. SS: soil suitability. Dotted blue lines were not included in statistical models owing to lack of data. See the text for details.

Our conceptual framework rests on the following assumptions. First, the morbidity and the lethality of human leptospirosis do not occur at the same rate across the Brazilian states. Furthermore, the temporal trends in morbidity and lethality vary across states. Regarding the causal determinants, our simplified assumption is as follows: Rainfall, with precipitation as proxy, might promote the formation and maintenance of stagnated water, increasing the soil suitability for *Leptospira* survival (Fig. 1a). This suitability is also affected by temperature (Fig. 1b) and other environmental variables (Fig. 1c). The soil moisture is a proxy of soil suitability and increases the risk of Leptospirosis morbidity (Fig. 1d). Environmental variables also affect the risk of Leptospirosis morbidity by means other than their effect on soil suitability (Fig. 1e,e’). Moreover, the higher the proportion of poor households in Brazilian states, the more common it is to find unfavorable socioeconomic contexts where waste management is inadequate or absent (Fig. 1f) and illiteracy is higher (Fig. 1g). In these poverty contexts, which differentially affect urban and rural areas, there is a lower ability to cope with floods, prevent the stagnation of water, and perform proper rodent (and other host species in rural areas) control. Thus, poverty affects Leptospirosis morbidity through waste management (Fig. 1i), illiteracy (Fig. 1i’) and through other socioeconomic factors (Fig. 1i”). An unknown fraction of Leptospirosis cases are misdiagnosed as dengue cases, an error that can lead to death of some patients owing to improper or delayed treatment (Fig. 1j,j’). To complete the causal network, we include biological agent and host determinants (Fig. 1k). Other febrile disease that may confuse the diagnostic of Leptospirosis were not considered because they have a localized occurrence (malaria is only present in the north region of Brazil), are almost absent (typhoid fever), or emerged or re-emerged recently and therefore there are no data for the entire studied period (yellow fever, zika virus and chikungunya).

### Statistical models

We performed exploratory analysis to verify data consistency and to obtain a preliminary idea of the association between covariates. Environmental covariates such as precipitation, temperature, and soil moisture were represented by more than one value (minimum, mean, maximum, standard deviation, range), and for each covariate, we selected the value that showed a stronger correlation with the leptospirosis relative risk (RR). This selection resulted in: minimum precipitation, maximum temperature, and minimum soil moisture.

All statistical models had a spatiotemporal architecture. The first set of models was used to model the RR of human leptospirosis cases, the second for human leptospirosis lethality, and the third to represent the conceptual framework of causality. Within each of these sets, models differed in their spatiotemporal architecture; in the last set, models also differed in the combination of covariates.

Given the *i* (*i* = *1*, *…*, *27*) states and *t (t* = *2000*, *…*, 2016) years, let *y*_*it*_ be the number of human leptospirosis cases in state *i* and year *t*, *P*_*it*_ be the human population at risk in state *i* and year *t*, and *E*_*it*_ be the yearly expected number of human leptospirosis cases in state *i* and year *t*, calculated by indirect standardization:

$${E}_{it}={P}_{it}\frac{{\sum }_{it}{y}_{it}}{{\sum }_{it}{P}_{it}}$$

We assumed that

$${y}_{it}|{\theta }_{it}\sim Poisson({E}_{it}{\theta }_{it}),$$

and *θ*_*it*_ is the state-year-specific RR. For lethality, *y*_*it*_ represented the number of deaths and *E*_*it*_ the number of cases. For the models of the causal network, *t* ranged from 2002 to 2014.

We evaluated two spatial, two temporal terms, and four spatiotemporal interactions. Let *υ*_*i*_ and *v*_*i*_ be the structured and unstructured spatial terms, respectively:

$${\upsilon }_{i}|{\upsilon }_{-i},\,{\tau }_{\varsigma }\varphi  \sim (\frac{1}{{\eta }_{\delta i}}\sum _{j\in {\eta }_{\delta i}}{\upsilon }_{j},\,\frac{1}{{\eta }_{\delta i}{\tau }_{\varsigma }\varphi })$$

$${v}_{i}\sim N(0,\,\frac{1}{{\tau }_{\varsigma }(1-\varphi )}),$$

where *τ*_ς_ is the marginal precision, *η*_*δi*_ is the number of neighbors of *i*, and *ϕ* measures the proportion of marginal spatial variance explained by υ. The overall spatial effect was given by^18^:

$$\varsigma =\frac{1}{\sqrt{{\tau }_{\varsigma }}}(\sqrt{1-\varphi }v+\sqrt{\varphi }\upsilon ),$$

This model is known as BYM2^18^. Let also *γ*_*t*_ and *ω*_*t*_ be the structured (random walk of first order: RW1) and unstructured temporal terms, respectively:

$${\gamma }_{t}|{\gamma }_{t-1} \sim N({\gamma }_{t-1},\,\frac{1}{{\tau }_{\gamma }})$$

$${\omega }_{t}\sim N(0,\,\frac{1}{{\tau }_{\omega }})$$

The spatiotemporal interactions *δ*_*it*_ were given by the Kronecker products *v*_*i*_ ⊗ *ω*_*t*_, *v*_*i*_ ⊗ *γ*_*t*_, *ω*_*t*_ ⊗ *υ*_*i*_ and *υ*_*i*_ ⊗ *γ*_*t*_^19^. *v*_*i*_ and *ω*_*t*_ were independent and identically distributed random variables (IID).

Considering the previous spatiotemporal terms, the intercept *β*_0_ and coefficients *β*_*p*_ (where *p* is the number of covariates) all distributed as $\beta  \sim N(0,\,\frac{1}{{\tau }_{\beta }})$, Table 2 presents the model nomenclature we will use hereafter. Note that for causal models of morbidity, models C1a and C1b estimated the effects of distal determinants (upper boxes in Fig. 1). Models C2a and C2b estimated the effect of soil, controlling for the effect of the distal determinants. Models C3a and C3b replaced poverty by the percentage of urban households, models C4a and C4b replaced poverty by the percentage of households with proper collection of waste, and models C5a and C5b replaced poverty by the number of illiterate persons. Poverty, waste management and illiteracy did not enter in the same model due to collinearity. The effect of soil moisture was not controlled when estimating the effect of precipitation and temperature because it was not a potential confounder; part of the effect of precipitation and temperature was mediated by the soil moisture. The difference between causality models with suffixes *a* and *b* is that models with suffix *a* had only unstructured terms *ω* and *v*, whereas those with suffix *b* had all spatial and temporal terms and the interaction between unstructured terms *δ*_*it*_ = *v*_*i*_ ⊗ *ω*_*t*_ (Table 2).

**Table 2:** Model nomenclature.

Model	Specification
RR	
RR01	*log*(*θ*_*it*_) = *β*_0_ + *v*_*i*_ + *ω*_*t*_
RR02	*log*(*θ*_*it*_) = *β*_0_ + *ς*_*i*_ + *ω*_*t*_ + *γ*_*t*_
RR1	*log*(*θ*_*it*_) = *β*_0_ + *ς*_*i*_ + *ω*_*t*_ + *γ*_*t*_ + *δ*_*it*_, *δ*_*it*_ = *v*_*i*_ ⊗ *ω*_*t*_
RR2	*log*(*θ*_*it*_) = *β*_0_ + *ς*_*i*_ + *ω*_*t*_ + *γ*_*t*_ + *δ*_*it*_, *δ*_*it*_ = *v*_*i*_ ⊗ *γ*_*t*_
RR3	*log*(*θ*_*it*_) = *β*_0_ + *ς*_*i*_ + *ω*_*t*_ + *γ*_*t*_ + *δ*_*it*_, *δ*_*it*_ = *ω*_*t*_ ⊗ *υ*_*i*_
RR4	*log*(*θ*_*it*_) = *β*_0_ + *ς*_*i*_ + *ω*_*t*_ + *γ*_*t*_ + *δ*_*it*_, *δ*_*it*_ = *γ*_*t*_ ⊗ *υ*_*i*_
Lethality	
L01	*log*(*θ*_*it*_) = *β*_0_ + *v*_*i*_ + *ω*_*t*_
L02	*log*(*θ*_*it*_) = *β*_0_ + *ς*_*i*_ + *ω*_*t*_ + *γ*_*t*_
L1	*log*(*θ*_*it*_) = *β*_0_ + *ς*_*i*_ + *ω*_*t*_ + *γ*_*t*_ + *δ*_*it*_, *δ*_*it*_ = *v*_*i*_ ⊗ *ω*_*t*_
L2	*log*(*θ*_*it*_) = *β*_0_ + *ς*_*i*_ + *ω*_*t*_ + *γ*_*t*_ + *δ*_*it*_, *δ*_*it*_ = *v*_*i*_ ⊗ *γ*_*t*_
L3	*log*(*θ*_*it*_) = *β*_0_ + *ς*_*i*_ + *ω*_*t*_ + *γ*_*t*_ + *δ*_*it*_, *δ*_*it*_ = *ω*_*t*_ ⊗ *υ*_*i*_
L4	*log*(*θ*_*it*_) = *β*_0_ + *ς*_*i*_ + *ω*_*t*_ + *γ*_*t*_ + *δ*_*it*_, *δ*_*it*_ = *γ*_*t*_ ⊗ *υ*_*i*_
Causality (RR)	*δ*_*it*_ = *v*_*i*_ ⊗ *ω*_*t*_
C1a	*log*(*θ*_*it*_) = *β*_0_ + *v*_*i*_ + *ω*_*t*_ + β_1_ precipitation_it_ + β_2_ temperature_it_ + β_3_ hh_poverty_it_
C1b	*log*(*θ*_*it*_) = *β*_0_ + *ς*_*i*_ + *ω*_*t*_ + *δ*_*it*_ + β_1_ precipitation_it_ + β_2_ temperature_it_ + β_3_ hh_poverty_it_
C2a	*log*(*θ*_*it*_) = *β*_0_ + *v*_*i*_ + *ω*_*t*_ + β_1_ precipitation_it_ + β_2_ temperature_it_ + β_3_ hh_poverty_it_ + β_4_ soil_it_
C2b	*log*(*θ*_*it*_) = *β*_0_ + *ς*_*i*_ + *ω*_*t*_ + *δ*_*it*_ + β_1_ precipitation_it_ + β_2_ temperature_it_ + β_3_ hh_poverty_it_ + β_4_ soil_it_
C3a	*log*(*θ*_*it*_) = *β*_0_ + *v*_*i*_ + *ω*_*t*_+ β_1_ precipitation_it_ + β_2_ temperature_it_ + β_3_ hh_urban_it_ + β_4_ soil_it_
C3b	*log*(*θ*_*it*_) = *β*_0_ + *ς*_*i*_ + *ω*_*t*_ + *δ*_*it*_ + β_1_ precipitation_it_ + β_2_ temperature_it_ + β_3_ hh_urban_it_ + β_4_ soil_it_
C4a	*log*(*θ*_*it*_) = *β*_0_ + *v*_*i*_ + *ω*_*t*_ + β_1_ precipitation_it_ + β_2_ temperature_it_ + β_3_ hh_collected_waste_it_ + β_4_ soil_it_
C4b	*log*(*θ*_*it*_) = *β*_0_ + *ς*_*i*_ + *ω*_*t*_ + *δ*_*it*_ + β_1_ precipitation_it_ + β_2_ temperature_it_ +β_3_ hh_collected_waste_itt_ + β_4_ soil_it_
C5a	*log*(*θ*_*it*_) = *β*_0_ + *v*_*i*_ + *ω*_*t*_ + β_1_ precipitation_it_ + β_2_ temperature_it_ + β_3_ illiterates_it_ + β_4_ soil_it_
C5b	*log*(*θ*_*it*_) = *β*_0_ + *ς*_*i*_ + *ω*_*t*_ + *δ*_*it*_ + β_1_ precipitation_it_ + β_2_ temperature_it_ + β_3_ illiterates_it_ + β_4_ soil_it_
Causality (lethality)	*δ*_*it*_ = *v*_*i*_ ⊗ *ω*_*t*_
C6a	*log*(*θ*_*it*_) = *β*_0_ + *v*_*i*_ + *ω*_*t*_ + β_1_ dengue_cases_it_
C6b	*log*(*θ*_*it*_) = *β*_0_ + *ς*_*i*_ + *ω*_*t*_ + *δ*_*it*_ + β_1_ dengue_cases_it_

The percentage of variability in random effects explained by covariates was measured by the relative change in the standard deviation (SD) of the random effects. For example, the effect of model C3b covariates in the random effect ς, taking RR1 as a model of reference, was given by (SD(ς_RR1_) − SD(ς_C3b_))/SD(ς_RR1_) * 100. The relative change was 100 if covariates explained all of the variability, 0 if they did not change the variability, and negative if they increased the variability.

The proportion of marginal variance explained by each component of the RR and lethality models was given by:

$${f}_{i}=1/n\,({{\tau }_{i}}^{-1}/({{\tau }_{\varsigma }}^{-1}+{{\tau }_{\gamma }}^{-1}+{{\tau }_{\omega }}^{-1})),\,i=\{\varsigma ,\,\gamma ,\,\omega \},$$

where *n* was the size (10,000) of samples drawn from the marginal posteriors *τ*_*ς*_, *τ*_*γ*_ and *τ*_*ρ*_. *f*_*ς*_ was further multiplied by ϕ and 1 − ϕ to obtain the proportion of variance explained by υ and *v*, respectively.

We characterized the RR and lethality of the correspondent best model in terms of fitted values, excess risk (RR_it_ > 1; lethality_it_ > average lethality), and a priority index (PI):

$$P{I}_{it}={x}_{it}ER({x}_{it})/max({x}_{it}ER({x}_{it}))\,100,$$

where *x*_*i*_ is the RR or lethality of the state *i*, ER is the excess risk, and *t* is the year; the PI was separately calculated for each year. The PI weights the RRs (or lethalities) by the corresponding excess risk, and the maximum value is scaled to 100. Other values are relative to that maximum. In addition, we calculated Pearson’s correlation coefficient between excess risk morbidity and lethality. All models were implemented using the Integrated Nested Laplace Approximation (INLA)^20^.

### Priors

The default priors used for this study were penalized complexity (PC) priors. Under the PC priors approach, model complexity is specified as the divergence between a flexible model and a base model^21^. In our case, the base model was characterized by *τ*_*i*_ = ∞, i = {ς, γ, ω, β} and *ϕ* = 0. In flexible models, *τ*_*i*_ < ∞ and *ϕ* > 0. The divergence was given by the unidirectional Kullback-Leibler divergence: $d({\tau }_{i})=\sqrt{KLD({\tau }_{i})},\,\,d(\varphi )=\sqrt{KLD(\varphi )}$ ^21^. The complexity was penalized by constant decay-rate, specified in terms of a type-2 Gumbel distribution, using the probability statements $Prob(1/\sqrt{{\tau }_{i}} > U=\alpha ),\,\,Prob(\varphi  < U=\alpha )$. These statements correspond to a decay rate equal to −*log*(*α*)/*U*^22^. As default priors we used $Prob(1/\sqrt{{\tau }_{i}} > 0.3/0.31=0.01)$ and *Prob*(*ϕ* < 0.5 = 0.7). In addition, we refitted the models with strong penalizing priors: $Prob(1/\sqrt{{\tau }_{i}} > 0.1/0.31=0.01)$ and *Prob*(*ϕ* < 0.1 = 0.9); and with vague priors: *Prob*(*ϕ* < 0.5 = 0.5), *τ*_*i*_ = *logGamma*(1,0.001).

### Model diagnostics

For each model, we calculated the deviance information criterion (DIC) and the posterior predictive p-value^22^. The predictive p-value is defined as *p*$({y}_{i}^{\ast }\le {y}_{i}/{\boldsymbol{y}})$, where ${y}_{i}^{\ast }$ is the predicted value of *y*_*i*_. Models with adequate fit have few p-values within the tail deciles^22^, so we calculated the proportion of p-values within the intervals [0, 0.1] and [0.9, 1]. We explored the sensitivity to priors comparing fitted, fixed, DIC, p-value and random posterior distributions. To evaluate the need for nonlinear models of covariate effects, we analyzed plots of each covariate against the model residuals. Plots with fitted locally weighted scatterplot smoothing (LOESS) trends around zero indicated that nonlinear effects were unnecessary. The results in the next section are based on the first set of priors, and Supplementary Figs 5 to 7 and Supplementary Table 3 present the results of the sensitivity analysis.

### Software and reproducibility

For the descriptive analysis and for data curation we used R package tidyverse 1.2.1^23^, tabulizer 0.1.24^24^, spdep 0.7–4^25^, sf 0.6–1^26^, ggsn 0.4.11^27^, gridExtra 2.3^28^, and animation 2.5^29^. All Bayesian models were implemented with INLA 17.12.01^20^, and model results were processed with INLAOutputs 1.4.10^30^. All data and code to reproduce the results are available in Supplementary-Analysis 1.

### Role of the funding source

The funder had no role in the collation of the data, development of the conceptual framework, analysis of data, interpretation of data, writing of the manuscript, or the decision to submit the paper for publication.

## Results

### Descriptive results

**Figure 2:**
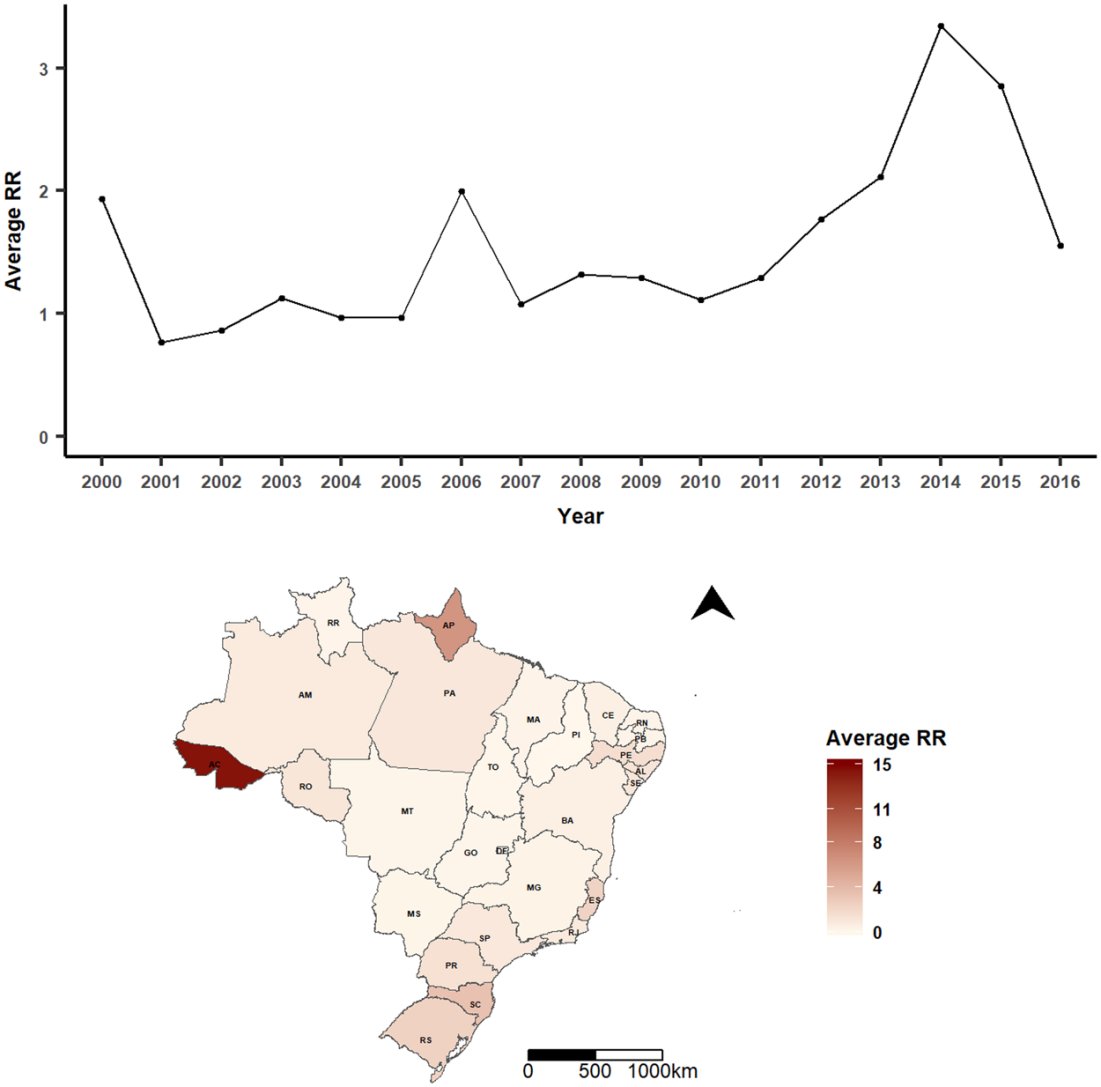
Spatiotemporal patterns of leptospirosis relative risk (RR) in Brazil, 2000–2016. Annual (top) and state averages (bottom). State’s identification: AC, Acre; AL, Alagoas; AP, Amapá; AM, Amazonas; BA, Bahia; CE, Ceará; DF, Distrito Federal; ES, Espírito Santo; GO, Goiás; MA, Maranhão; MT, Mato Grosso; MS, Mato Grosso do Sul; MG, Minas Gerais; PR, Paraná; PB, Paraíba; PA, Pará; PE, Pernambuco; PI, Piauí; RJ, Rio de Janeiro; RN, Rio Grande do Norte; RS, Rio Grande do Sul; RO, Rondônia; RR, Roraima; SC, Santa Catarina; SE, Sergipe; SP, São Paulo; TO, Tocantins.

**Figure 3:**
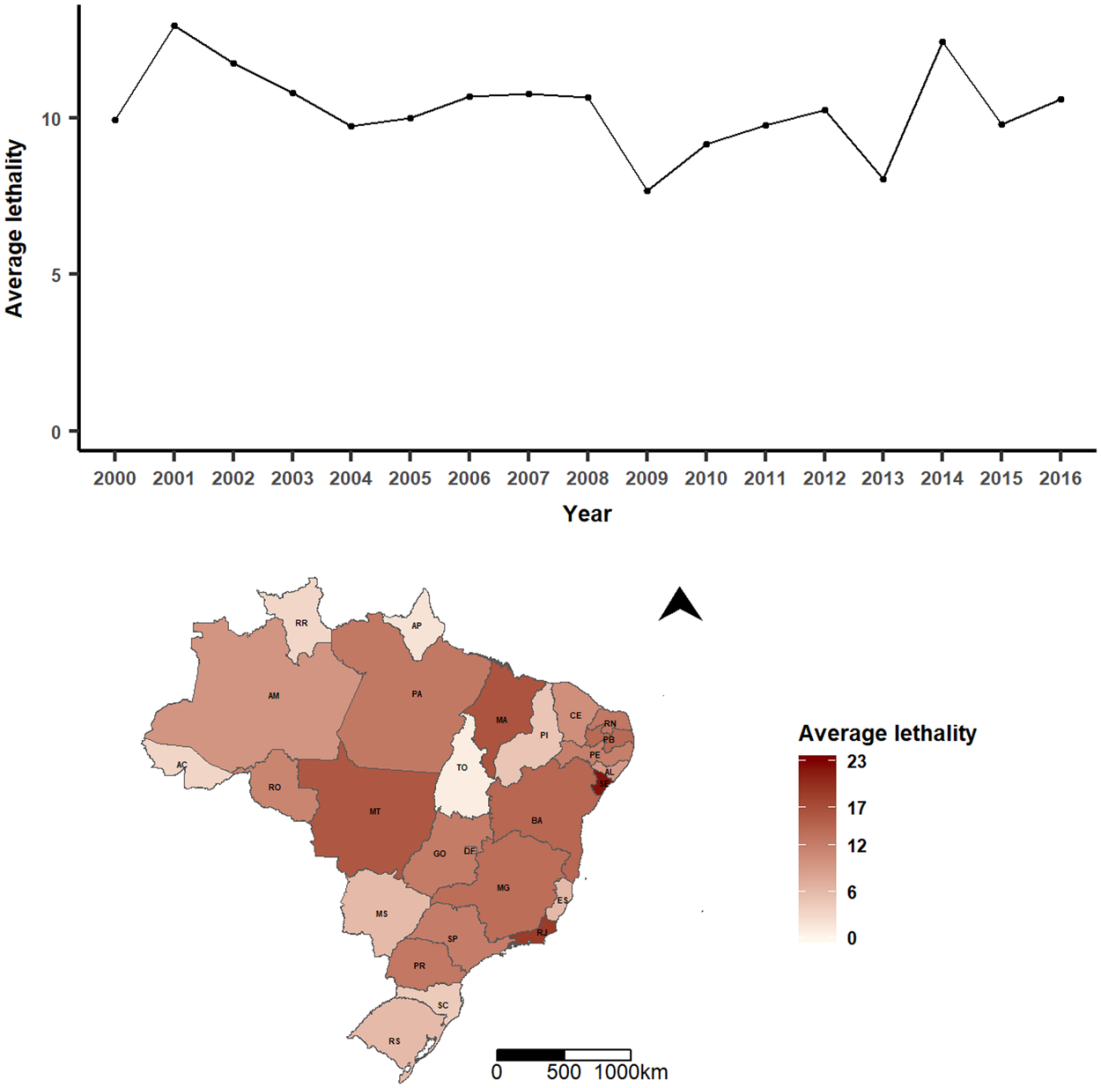
Spatiotemporal patterns of leptospirosis lethality in Brazil, 2000–2016. Annual (top) and state averages (bottom). State’s identification: AC, Acre; AL, Alagoas; AP, Amapá; AM, Amazonas; BA, Bahia; CE, Ceará; DF, Distrito Federal; ES, Espírito Santo; GO, Goiás; MA, Maranhão; MT, Mato Grosso; MS, Mato Grosso do Sul; MG, Minas Gerais; PR, Paraná; PB, Paraíba; PA, Pará; PE, Pernambuco; PI, Piauí; RJ, Rio de Janeiro; RN, Rio Grande do Norte; RS, Rio Grande do Sul; RO, Rondônia; RR, Roraima; SC, Santa Catarina; SE, Sergipe; SP, São Paulo; TO, Tocantins.

From January 2000 to December 2016, a total of 63,302 cases and 6,064 lethal leptospirosis notifications were registered by the SINAN, Brazil^14^. The total annual case counts ranged from 2,769 in 2002 to 4,874 in 2011, and the number of deaths ranged from 234 in 2016 to 436 in 2001. The minimum annual RR average was 0.79 (2001), while the maximum was 3.34 in 2014 (Fig. 2). The spatial distribution of the RR average is shown in Fig. 2. The minimum average lethality per one hundred thousand cases was 3.34 (2009); the maximum was 12.94 in 2001 (Fig. 3). The spatial distribution of the average lethality rates from 2000 to 2016 is mapped in Fig. 3 (see Supplementary-Table 1 and Supplementary Figs 1 and 2 for values of each state annual RR and lethality, respectively).

In addition, all covariates used in the conceptual framework of causality were described and mapped to show each spatiotemporal distribution (Supplementary-Figs 8 to 14).

### Spatiotemporal RR and lethality models

In RR models, the spatial and temporal structured effects improved the model fitting, but the better spatiotemporal interaction term *δ*_*it*_ was among unstructured effects (Table 3). In L models, the improvements caused by structured effects were lower and the best spatiotemporal interaction term *δ*_*it*_ was among the unstructured spatial effect and the structural temporal effect. In RR1, the best of the RR models, the unstructured spatial effect and the spatiotemporal interaction were the major contributors to the explained variance. In L2, the best of the L models, the spatiotemporal interaction was the major contributor to the explained variance, followed by the unstructured spatial effect (Table 4). The temporal effects were more relevant in L2 than in RR1 (Table 4).

**Table 3:** Model diagnostics for spatiotemporal relative risk (RR) and lethality models. p-value: percentage of values in the lower and upper deciles (lower, upper).

Model	DIC	p-value	Model	DIC	p-value
RR01	18,279.12	(52.9, 38.1)	L01	2178.60	(47.5, 35.3)
RR02	18,282.31	(52.9, 38.1)	L02	2,176.11	(47.3, 34.6)
RR1	3,403.36	(7.2, 0.2)	L1	2,088.19	(34.9, 19.8)
RR2	3,408.14	(8.7, 1.3)	L2	2,042.11	(35.7, 19.4)
RR3	3,412.61	(8.7, 1.3)	L3	2,108.19	(39.0, 23.3)
RR4	3,415.16	(10.7, 2.0)	L4	2,047.10	(38.3, 21.1)

**Table 4:** Percent contribution of each model’s random effect to the variance explained by the model.

Random effect	RR1 model	L2 model
ν	66.7	26.4
υ	11.2	11.8
ω	0.1	2.0
γ	0.7	7.7
δ	21.3	52.1

**Figure 4:**
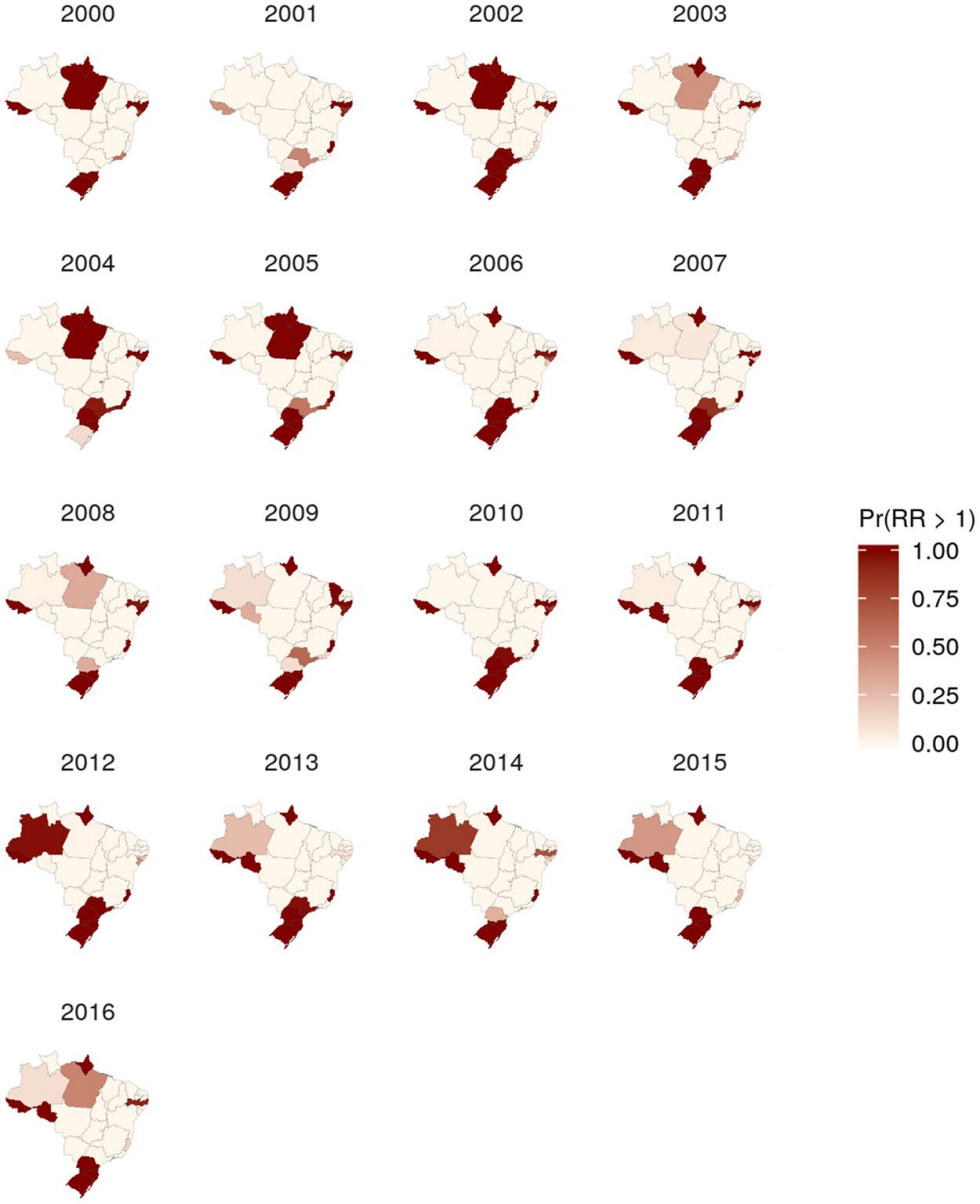
Spatiotemporal pattern of the posterior mean excess risk morbidity, predicted by the RR1 model.

**Figure 5:**
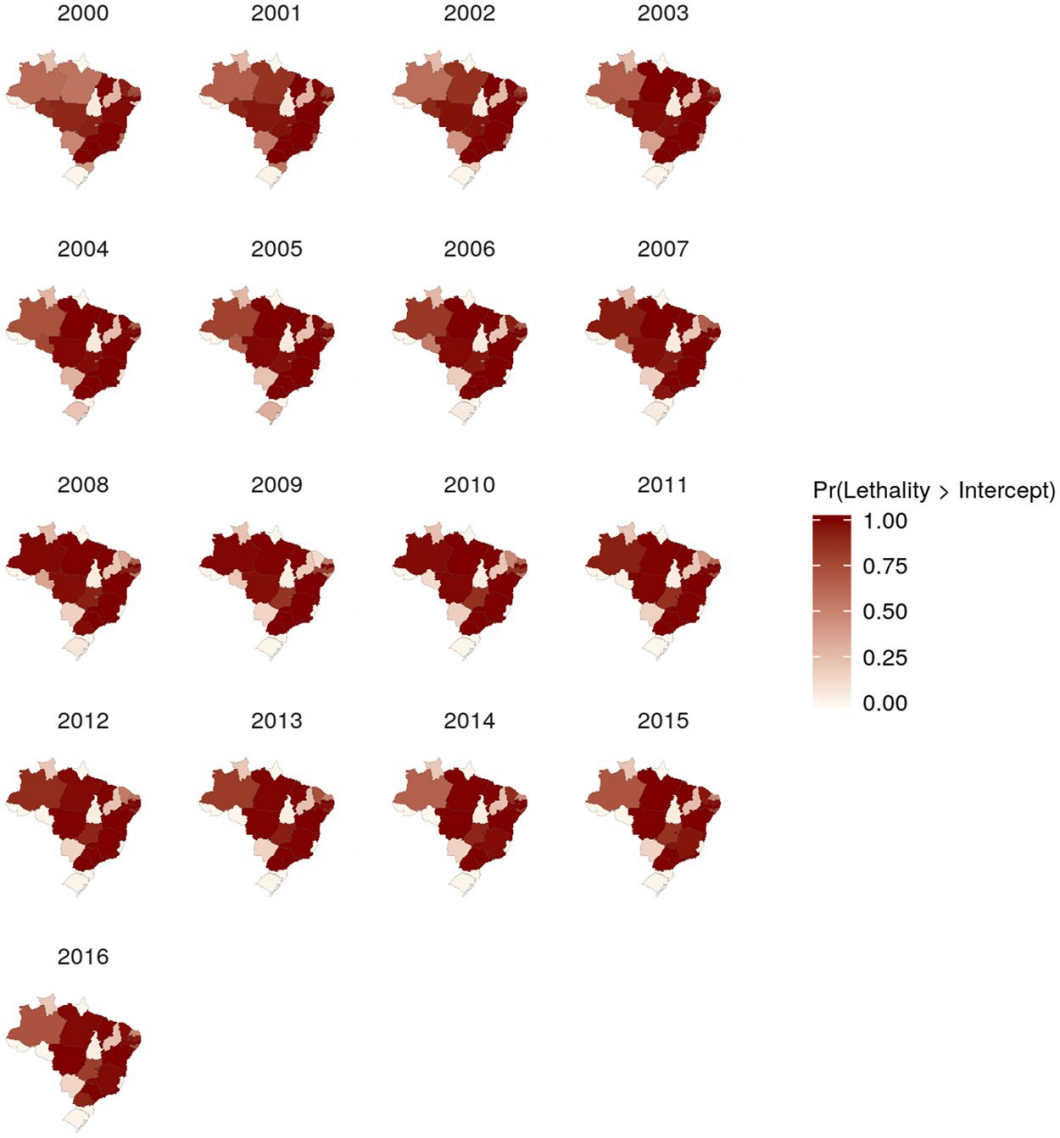
Spatiotemporal pattern of the posterior mean excess risk lethality (per 100,000 cases), predicted by the L2 model.

**Figure 6:**
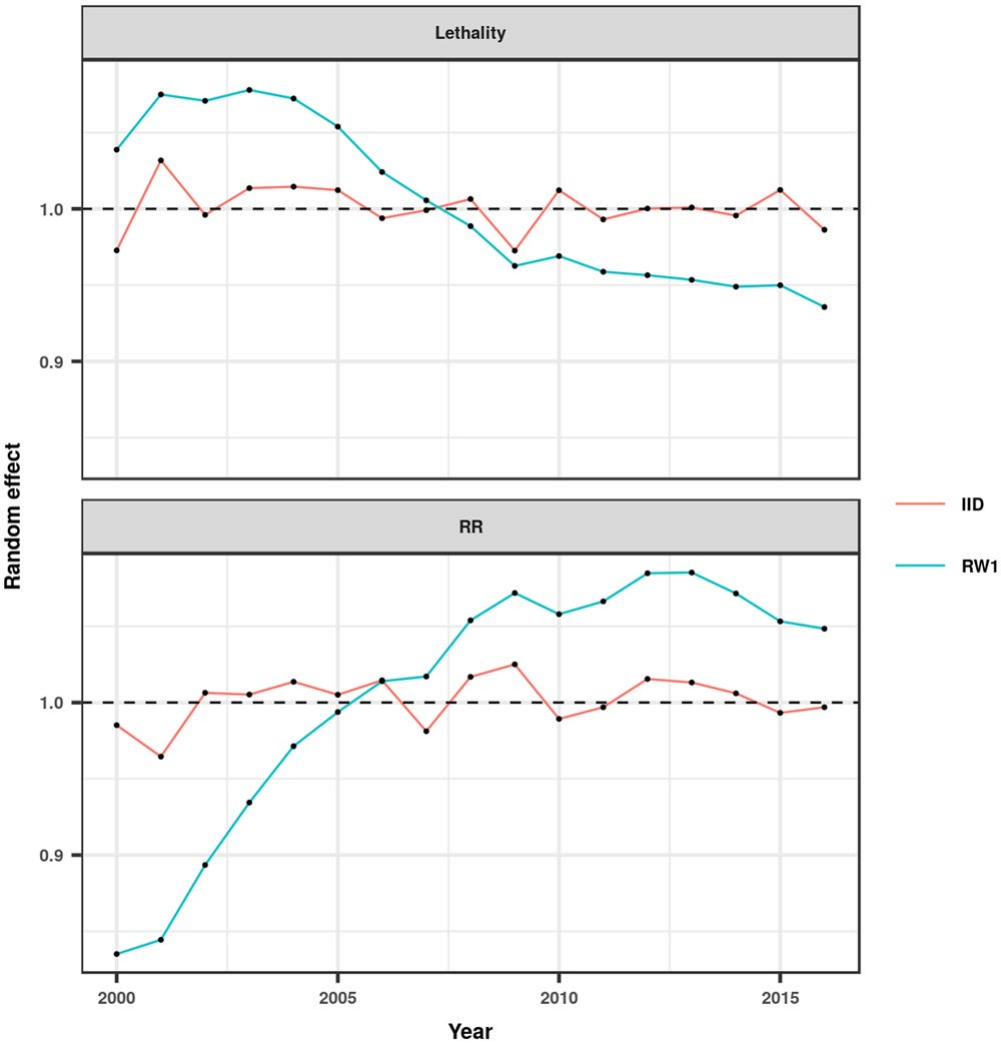
Spatiotemporal pattern of the posterior mean temporal trends of morbidity (bottom) and lethality (top), predicted by the RR1 and L2 models, respectively. IID: independent and identically distributed unstructured temporal effect, RW1: structured temporal effect distributed as a random walk of first order.

**Figure 7:**
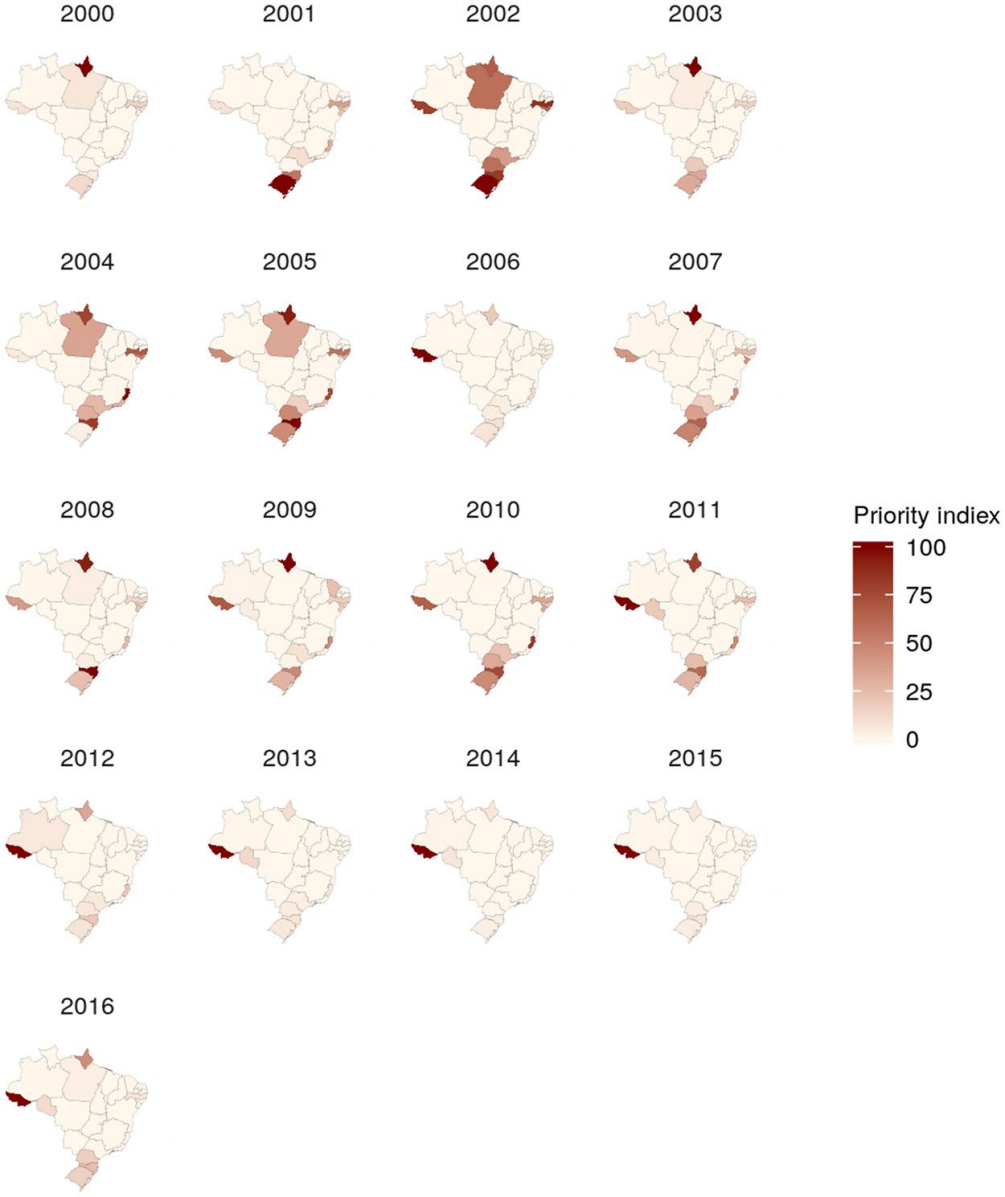
Spatiotemporal pattern of the morbidity priority index.

**Figure 8:**
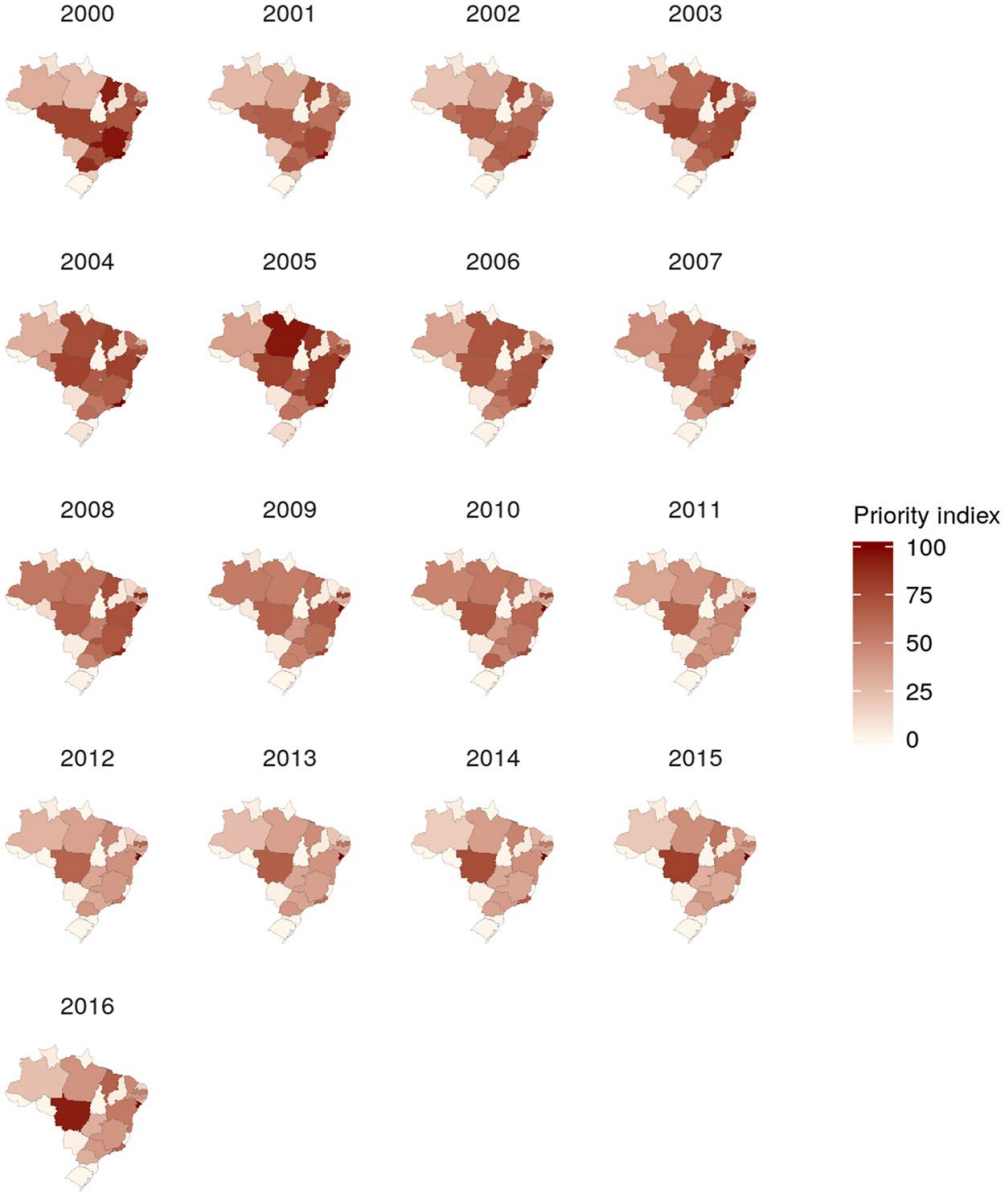
Spatiotemporal pattern of the lethality priority index.

The spatiotemporal patterns of the calculated posterior means were heterogeneous, especially for morbidity (Figs 4 and 5, Supplementary Figs 1 to 4, and Supplementary-Anim 1 and Supplementary-Anim 2). The morbidity and lethality patterns tended to be opposed, as demonstrated by the negative correlation between their posterior means for the excess risk (Pearson’s correlation coefficient = −0.27, credible interval (CI) 95% = −0.36, −0.19). The structured temporal trend increased for morbidity and decreased for lethality (Fig. 6). The morbidity PI of the last five years was particularly high in the Acre State (Fig. 7); the lethality PI was less variable (Fig. 8). State-year PIs are also described in Supplementary-Table 2.

### Causality models

Based on models with suffix *a*, soil moisture was the strongest risk factor and an increase in this property, equivalent to one SD, increased the risk of leptospirosis morbidity by 55.9%. Poverty and precipitation were the other risk factors, with effect sizes of 7.7% and 4.5%, respectively (Table 5). An increase in one SD in the percentage of urban households decreased the risk by 14.6%, while an equivalent increase in the percentage of households with proper collection of waste decreased the risk by 6% (Table 5). The temperature was a preventive factor, while the number of illiterate individuals had a credible interval compatible with the absence of effect (Table 5). The reported effects of soil moisture were calculated controlling for poverty. These effects were similar and qualitatively equivalent when poverty was replaced by the percentage of urban households, the percentage of households with proper collection of waste or with the number of illiterates individuals. The number of dengue cases had no effect on lethality: RR = 1⋅006 (0.944–1.038). The plots of covariates against model residuals did not show nonlinear trends (Supplementary-Fig. 15). In models with suffix *b*, only soil moisture and temperature had credible intervals excluding the absence of effect. Models C3a and C3b had the lowest DIC in within each group of models (Table 6). As those models had the same spatiotemporal architecture as RR1, we were interested in calculating the percentage of variability in the random effects explained by covariates. When compared to RR1, all these models reduced the variability of the global spatial effect and of the spatiotemporal interaction; the variability of some temporal effects increased (Table 7).

**Table 5:** Effect of covariates on the relative risk (RR) of leptospirosis morbidity.

Covariate	RR (95% CI)	Model
Precipitation (average minimum)	1.045 (1.023–1.067) 1.020 (0.893–1.159)	C1a C1b
Temperature (average maximum)	0.541 (0.503–0.581) 0.441 (0.321–0.590)	C1a C1b
Poverty	1.077 (1.031–1.126) 1.036 (0.858–1.286)	C2a C2b
Soil moisture	1.559 (1.435–1.691) 1.49 (1.08–1.99)	C2a C2b
Households (%) in urban areas	0.854 (0.829–0.880) 0.892 (0.782–1.013)	C3a C3b
Households (%) with proper collection of waste	0.939 (0.909–0.970) 1.045 (0.899–1.204)	C4a C4b
Number of illiterate individuals	0.941 (0.879–1.004) 1.509 (0.818–1.361)	C5a C5b

CI: Credible Interval.

**Table 6:** Comparison of causality models.

Model	DIC	Model	DIC
C1a	10377.97	C1b	2615.77
C2a	10270.89	C2b	2616.64
C3a	10161.41	C3b	2615.11
C4a	10268.09	C4b	2616.66
C5a	10277.08	C5b	2616.50

**Table 7:** Percentage of variability in random effects explained by covariates.

Random effect	Model				
	C1b	C2b	C3b	C4b	C5b
ς	19.4	25.4	25.3	24.6	25.0
ω	−47.1	−11.8	−13.7	−29.4	0.0
γ	25.5	24.5	−2.0	29.4	12.7
δ	170	17.1	17.3	17.3	17.1

Model of reference: RR1. Negative values indicate an increase in the variability.

## Discussion

The morbidity and lethality of human leptospirosis presented different spatiotemporal patterns and did not occur systematically in the same states for the entire period. Five out of the seven hypothesized causal network covariates had the expected direction of association and explained a fraction of the spatial and spatiotemporal interaction variability. The outputs of the analyses presented here highlighted which regions would benefit from reinforcement of disease control, surveillance, and funding strategies. The approach developed in this study also offered a complementary mapping approach, including a risk-based PI that ranked the states in order for prioritization, generating a valuable evidence base to guide and improve leptospirosis surveillance, control, and elimination planning.

The spatiotemporal dynamics of human leptospirosis were explored in the entire national territories of both China^31^ and Colombia^32^, in China from 2005 to 2015 and in Colombia from 2007 to 2016. In China, the crude rates of morbidity and mortality decreased over this period, and the temporal trends were modeled assuming linearity. Crude rates were presented at the province levels and were higher in the south of the country. In Colombia, six spatiotemporal high-risk clusters of morbidity were identified using scan statistics. There was an epidemic peak over a period of a few months, but no clear trend during the entire period examined. Our study used spatiotemporal models appropriate for crude rates that can be misleading owing to sparsity or small values, as is the case with the human leptospirosis rates. The models also allowed us to explore nonlinear trends over a period of 17 years. The spatiotemporal variation we observed was expected, assuming that many of its causes vary across space and time. This variation in disease occurrence can be detected at different scales, as demonstrated by our study and other studies restricted to smaller areas and shorter periods^13,33^. However, detection of spatiotemporal interactions might be more dependent on scale and methodology. A four-year prospective study in a Brazilian slum^13^ detected spatial and temporal variations but was unable to identify spatiotemporal interactions: although incidence varied across years, the spatial pattern was the same, and specific hot-spots consistently had higher risk of transmission during the study years. In contrast, we explicitly modeled spatiotemporal interactions over 17 years and these had considerable explanatory power, more so for lethality (Table 4); in other words, the hot spots were not always the same and the temporal trend varied across states (see Supplementary Figs 3 and 4). However, the interpretation of the lethality models requires caution due its limited fit to the data suggested by the fraction of posterior predictive p-values in the tail deciles (Table 3)^22^.

The conceptual framework assumed that environmental and socioeconomic determinants affect morbidity but not lethality (Fig. 1). These determinants had their own spatiotemporal patterns, and they explained a fraction of the spatiotemporal variability in morbidity. The spatiotemporal pattern of lethality was less variable than that observed for morbidity, probably because it in fact was not affected, or at least was less affected, by environmental and socioeconomic determinants. Moreover, sub-notification, especially for cases in the early stage of disease, could have contributed to the spatiotemporal variability of morbidity and lethality, but to the former to a greater extent. This could have been related to the negative correlation between the spatiotemporal patterns of morbidity and lethaliy. If one assumes that there are more sources of variation for morbidity and that sub-notification is more influential on morbidity, one would expect to find, as we did, greater heterogeneity in spatiotemporal patterns of morbidity.

Multiple causal factors establish complex interactions that determine the risk of transmission of human leptospirosis. Although the causal network is not fully understood, ecological^10,34^, multilevel^13,33^, and individual-level^35,36^ studies have identified environmental and socioeconomic variables that consistently act as risk factors. We decided to synthesize some of these previous findings in an explicit conceptual model of the causal network of human leptospirosis, with a focus on variables readily available, and indexed by spatial and temporal units. We chose the state and the year as spatial and temporal units, respectively, because these were the units in which socioeconomic variables of interest were available, and because they allowed us to test the causal hypothesis at the same resolution that supported our spatiotemporal dynamics findings of morbidity and lethality. These units implied the aggregation of data over large areas and periods, which could have diluted or reversed the direction of the associations under study. However, five of the seven covariates had effects consistent with the literature reports, as described below. Furthermore, covariates explained a substantial fraction of the spatial and spatiotemporal interaction variability.

Most reports of leptospirosis include exposure to contaminated soil and water but not direct contact with animals, so it is thought that the most common method of transmission is mediated by contaminated soil and water^33,37,38^. *Leptospira* is recurrent in soil with a moisture content > 20%^39^ and survives in temperatures ranging from 4 to 40 °C^40^. These parameters entail a wide range of environments and offer little guidance for surveillance. However, individual-level studies – as concluded by a systematic review – nearly always identify as risk factors water-related activities and exposure to floods and rainfall^11^. An ecological study also found that risk increases with increased rainfall^41^, and we observed that the involvement of rainfall as a risk factor is also verifiable on large spatiotemporal scales. The soil type might also influence the occurrence of human leptospirosis, according to ecological studies concluding that heavy sabulous clay soils^34^ and Neossolo Litolítico soils^10^ have a positive association with the occurrence of human leptospirosis. Sabulous clay soils allow water to inundate the soil when inclination is favorable (as in the study of Rodd *et al*.^34^) and Neossolo Litolítico soils have a low drainage capacity. Thus, these types of soils might have a higher moisture content suitable for the survival of *Leptospira*. Our results support this rationale and provide ecological evidence based on a variable (soil available water capacity)^15^ that is a more direct measure of the available water that can be stored in the soil. Higher temperatures seem to increase the risk of human leptospirosis^42^, but we found the opposite. A recent German study in muskrats also found an inverse association between leptospirosis and maximum temperatures^43^. One possible explanation for these findings may be the fact that warm temperatures deplete the moisture in the environment, which would reduce the chances for the survival of *Leptospira* outside its host^5^. This contradicting evidence regarding the relationship between temperature and leptospirosis morbidity should clarified by modeling individual outcomes under multilevel approaches, using covariates at finer spatial and temporal scale (e.g., at the municipality and month levels).

Poverty has been considered a risk factor for human leptospirosis^13^, and we hypothesized that under conditions of poverty, illiteracy is higher, waste management is inadequate, and there is a lower ability to cope with floods, prevent the stagnation of water, and ensure proper rodent control. The hypothesized relationship between poverty, illiteracy, and waste management was verified among covariates. As expected, poverty was a risk factor and better waste management a protective factor. Urbanization was also a protective factor, possibly owing to its negative association with poverty and its positive association with waste management. Illiteracy was not a direct risk factor, possibly because its effect was confounded by poverty. Illiteracy, waste management and poverty did not enter in the same model due to collinearity. It should be noted that disorganized urbanization can promote leptospirosis transmission, and living in slums is a risk factor^13^, but these findings do not necessarily mean that urban areas are at greater risk than rural ones. In fact, our results are not the first to point to rural areas as associated with greater risk^44^.

The covariates had no effect in models with structured random effects (causality models with suffix *b*), perhaps because they had a spatiotemporal pattern masked by the structured effects. It is possible that the percentage of variability in spatial and spatiotemporal random effects explained by covariates resulted from this masking effect, which has been documented in simulation and empirical studies based on spatial models with structured effects^45,46^. Covariates explained a fraction of the variability of most random effects. Some models with covariates increased the variability in temporal random effects, but it should be remembered that ω and γ explained only 0.1% and 0.7% of the model variability. Therefore, these increases were negligible.

Infectious diseases in general are often sensitive to variability on a fine spatiotemporal scale that was not well represented in our study. This is problematic because the dynamics of infectious diseases may be particularly sensitive to extremes or variability removed in the aggregation of the data^13^. Therefore, our findings should be complemented by more detailed characterizations in order to guide interventions within the states.

The interpretability of numeric information can be conditioned by the format in which the information is presented^47^. The PI used is a risk-based percentage scale that ranks units of analysis and measures the distance between them. It might facilitate the communication of spatiotemporal risk predictions, especially to stakeholders lacking background to interpret concepts such as spatial RR and excess risk. The PI suggests the order in which the units of analysis should be prioritized and how different the prioritization assigned to them should be. In terms of morbidity, it was clear that the prioritization should be focused in a couple of states, mainly in Acre (see Supplementary Table 2). In terms of lethality, the allocation of resources need not be as asymmetric, but nonetheless there was a prioritization order (see Supplementary-Table 2 for each state-year PI).

Spatiotemporal patterns of morbidity were more heterogeneous than those of lethality, probably because there are more causal determinants acting on morbidity. Sub-notification might have contributed to these patterns, and we urge improvements in leptospirosis reporting in all states. The causal model synthesized previous findings regarding the contextual determinants of leptospirosis, most of its components agreed with observed data, and it serves as a guide for future research on causal determinants. The PI might facilitate communication with decision makers and the general public, showing which states are the most problematic, and to what extent they should be prioritized.

## Electronic supplementary material

Anim. 1

Anim. 2

Anim. 3

Anim. 4

Supplementary-material

Supplementary-Analysis

## Data Availability

All results can be reproduced using the data and code to available as Supplementary data.
