# Author Correction: Spatiotemporal dynamics and risk factors for human Leptospirosis in Brazil

**DOI:** 10.1038/s41598-019-42747-0

**Published:** 2019-05-01

**Authors:** Oswaldo Santos Baquero, Gustavo Machado

**Affiliations:** 1 Department of Preventive Veterinary Medicine and Animal Health, School of Veterinary Medicine and Animal Science, University of São Paulo, Av. Prof. Orlando Marques de Paiva, 87, Cidade Universitária, São Paulo, SP CEP: 05508-270 Brazil; 2 Department of Population Health and Pathobiology, College of Veterinary Medicine, North Carolina State University, 1060 William Moore Drive, Raleigh, NC 27607 USA

Correction to: *Scientific Reports* 10.1038/s41598-018-33381-3, published online 11 October 2018

This Article contains an incomplete equation in the Methods section, under the subheading ‘Statistical models’.

“The proportion of marginal variance explained by each component of the RR and lethality models was given by:

$${f}_{i}=1/n\,({\tau }_{i}^{-1}/({\tau }_{\varsigma }^{-1}+{\tau }_{\gamma }^{-1}+{\tau }_{\omega }^{-1})),\,i=\{\varsigma ,\gamma ,\omega \},$$

where n was the size (10,000) of samples drawn from the marginal posteriors τ_ς_, τ_γ_ and τ_ρ_. f_ς_ was further multiplied by φ and 1 − φ to obtain the proportion of variance explained by υ and v, respectively”.

should read:

“The proportion of marginal variance explained by each component of the RR and lethality models was given by:

$${f}_{i}=\frac{1}{n}\,\,\sum _{1={\rm{k}}}^{{\rm{n}}}(\frac{{\tau }_{ki}^{-1}}{{\tau }_{k\varsigma }^{-1}+{\tau }_{k\gamma }^{-1}+{\tau }_{k\omega }^{-1}}),\,i=\{\varsigma ,\gamma ,\omega \},$$

where n was the sample size (10,000) of samples drawn from the marginal posteriors τ_ς_, τ_γ_ and τ_ρ_. f_ς_ was further multiplied by φ and 1 − φ to obtain the proportion of variance explained by υ and v, respectively”.

