# Supplementary figures and images for: Spatiotemporal dynamics and risk factors for human Leptospirosis in Brazil
Source: Sci Rep. 2018 Oct 11;8:15170. doi: 10.1038/s41598-018-33381-3 (PMC6181921; doi:10.1038/s41598-018-33381-3)

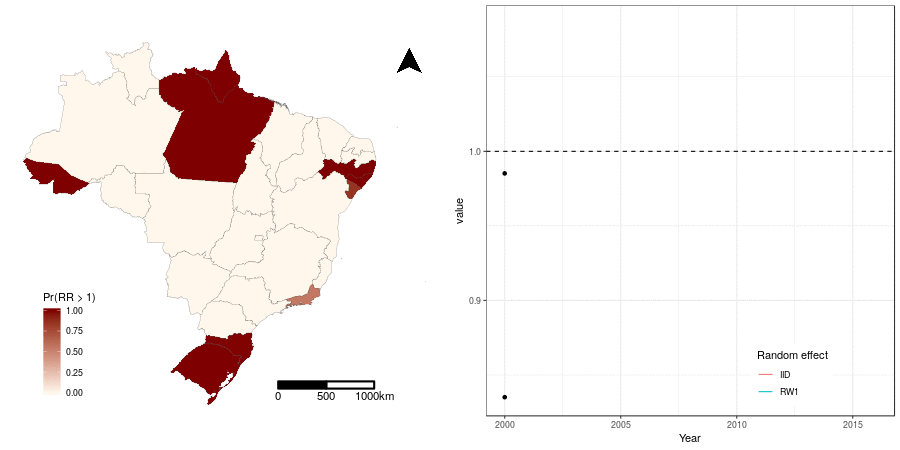

Supplement: Supplementary file 1 — Anim. 1 [file 41598_2018_33381_MOESM1_ESM.gif]

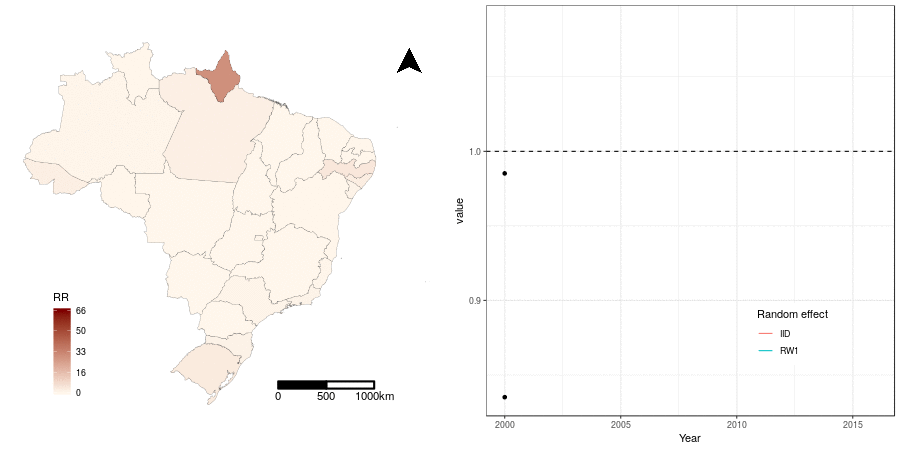

Supplement: Supplementary file 2 — Anim. 2 [file 41598_2018_33381_MOESM2_ESM.gif]

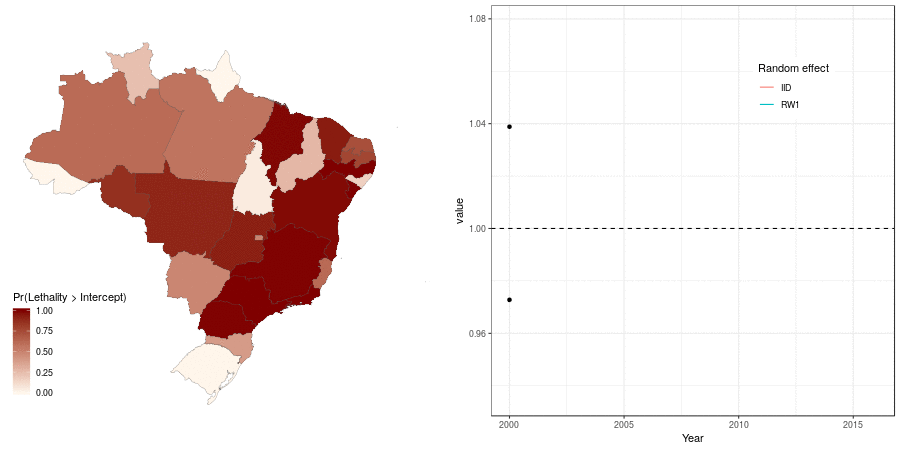

Supplement: Supplementary file 3 — Anim. 3 [file 41598_2018_33381_MOESM3_ESM.gif]

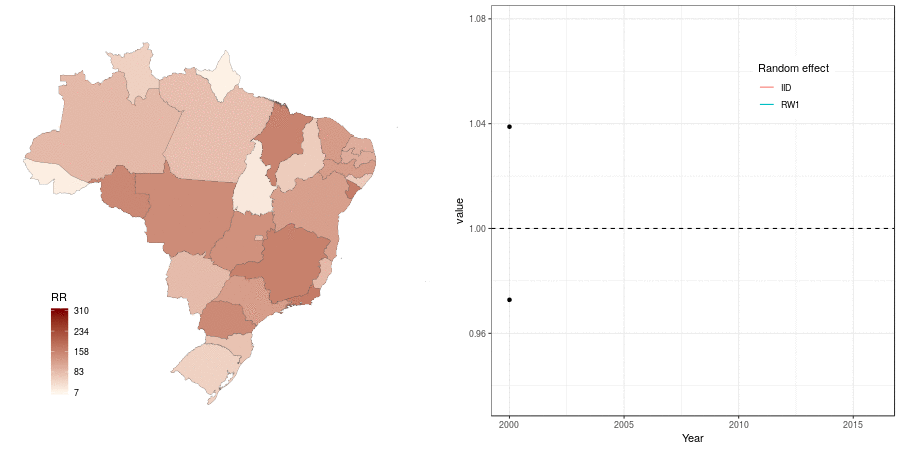

Supplement: Supplementary file 4 — Anim. 4 [file 41598_2018_33381_MOESM4_ESM.gif]
