# Supplementary material for: Spatiotemporal dynamics and risk factors for human Leptospirosis in Brazil
Source: Sci Rep. 2018 Oct 11;8:15170. doi: 10.1038/s41598-018-33381-3 (PMC6181921; doi:10.1038/s41598-018-33381-3)
Supplement: Supplementary file 5 — Supplementary-material [file 41598_2018_33381_MOESM5_ESM.pdf]

Spatiotemporal dynamics and risk factors for human Leptospirosis in Brazil

Oswaldo Santos Baquero, PhD [1], Gustavo Machado, PhD [2]

[1] Department of Preventive Veterinary Medicine and Animal Health, School of Veterinary Medicine and Animal Science, University of São Paulo, Av. Prof. Orlando Marques de Paiva, 87, Cidade Universitária, São Paulo, SP CEP: 05508-270, Brazil
[2] Department of Population Health and Pathobiology, College of Veterinary Medicine, North Carolina State University, 1060 William Moore Drive Raleigh, NC, 27607. USA.

Supplementary material

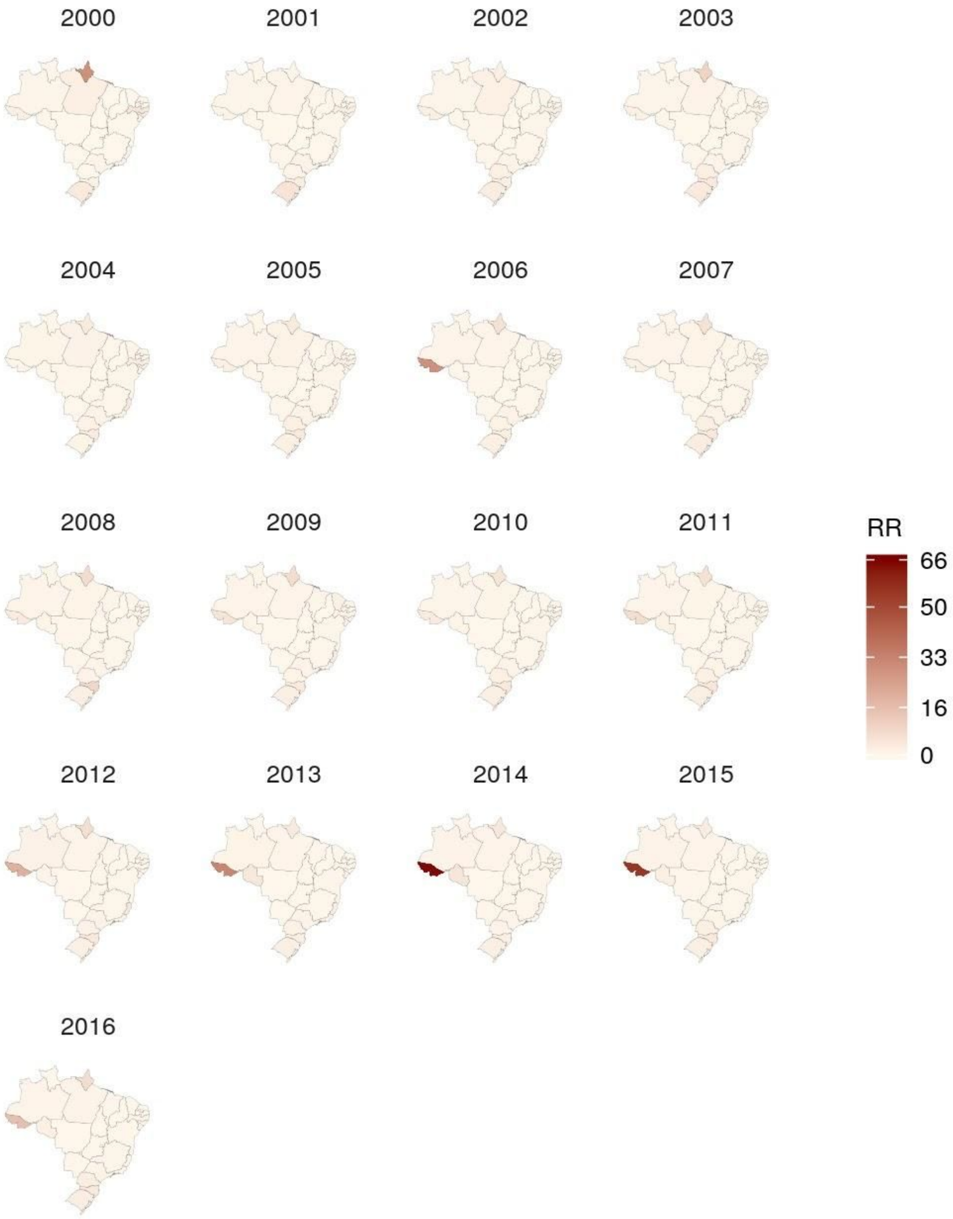

Fig. 1. Posterior mean Relative Risk (RR) of leptospirosis morbidity, Brazil. 2000–2016.

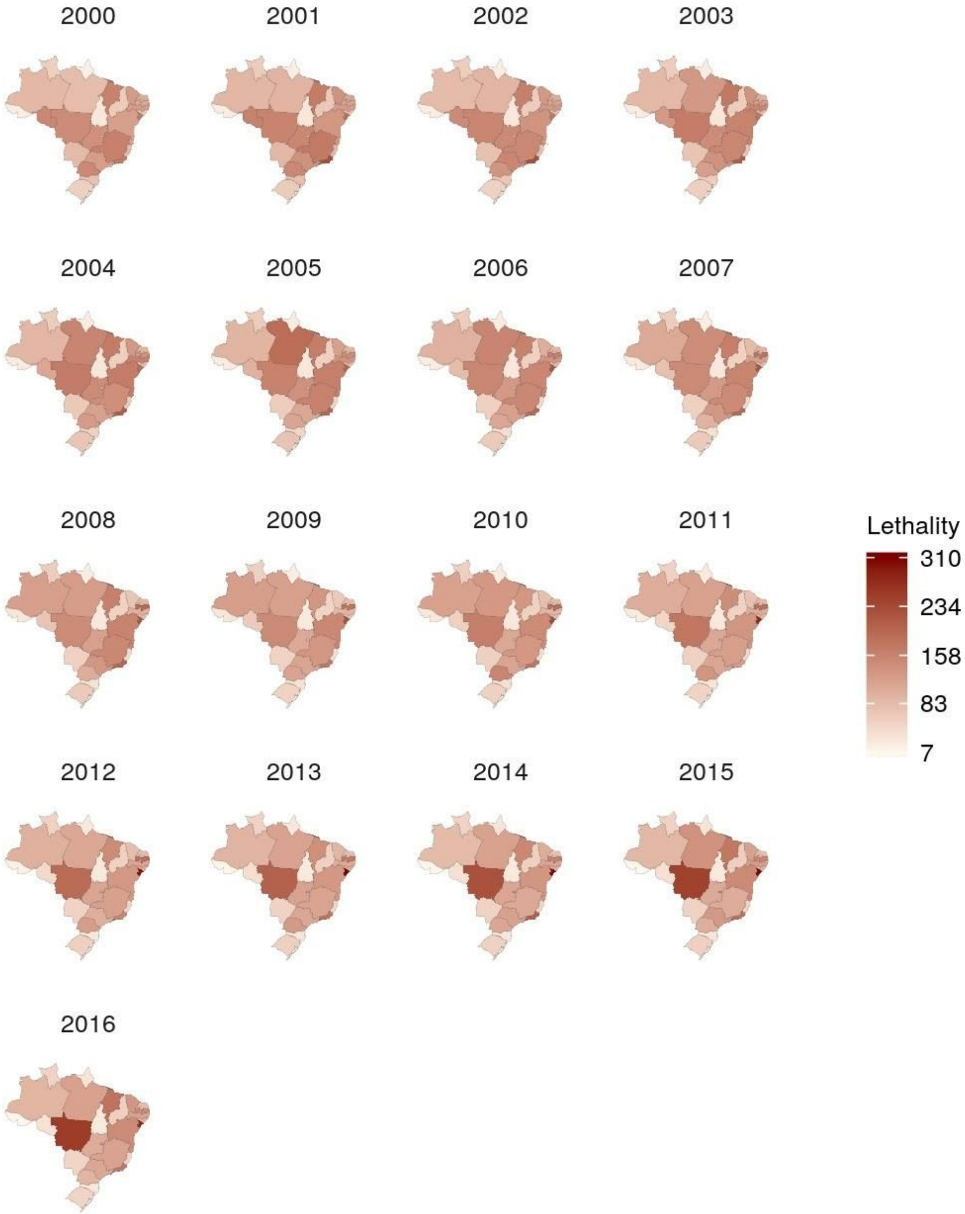

Fig. 2. Posterior mean leptospirosis lethality, Brazil. 2000–2016.

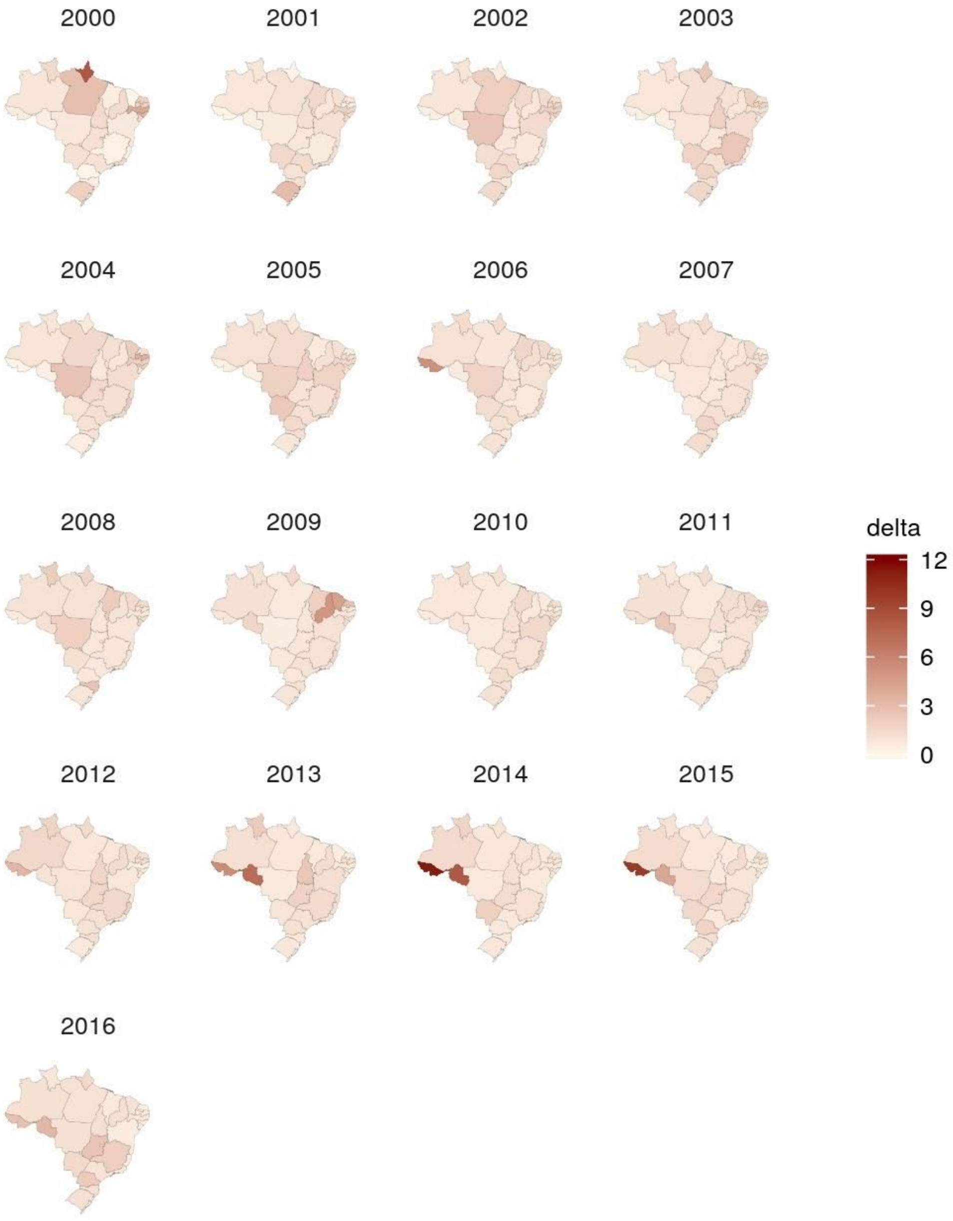

Fig. 3. Posterior mean spatiotemporal random effect of leptospirosis morbidity, Brazil. 2000–2016.

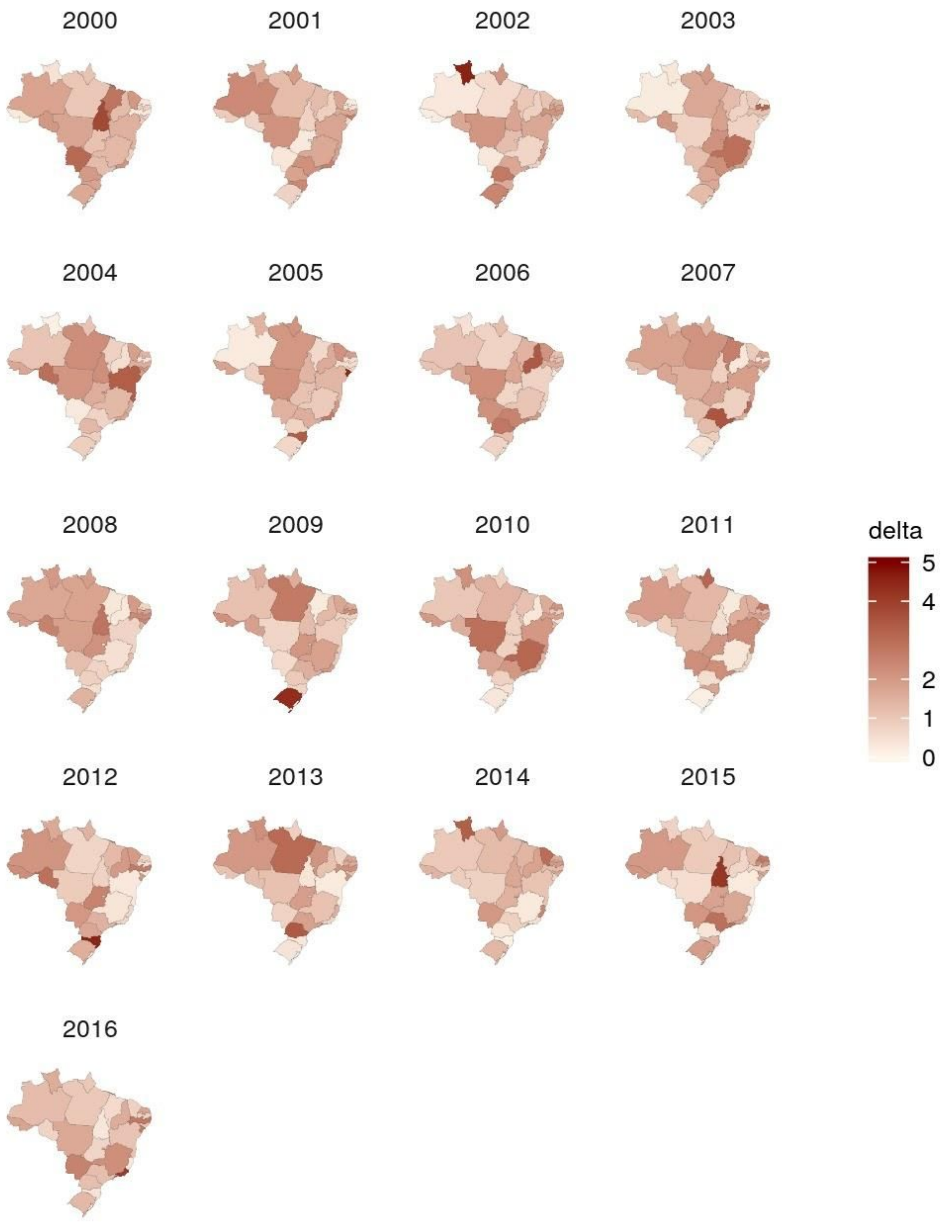

Fig. 4. Posterior mean spatiotemporal random effect of leptospirosis lethality, Brazil. 2000–2016.

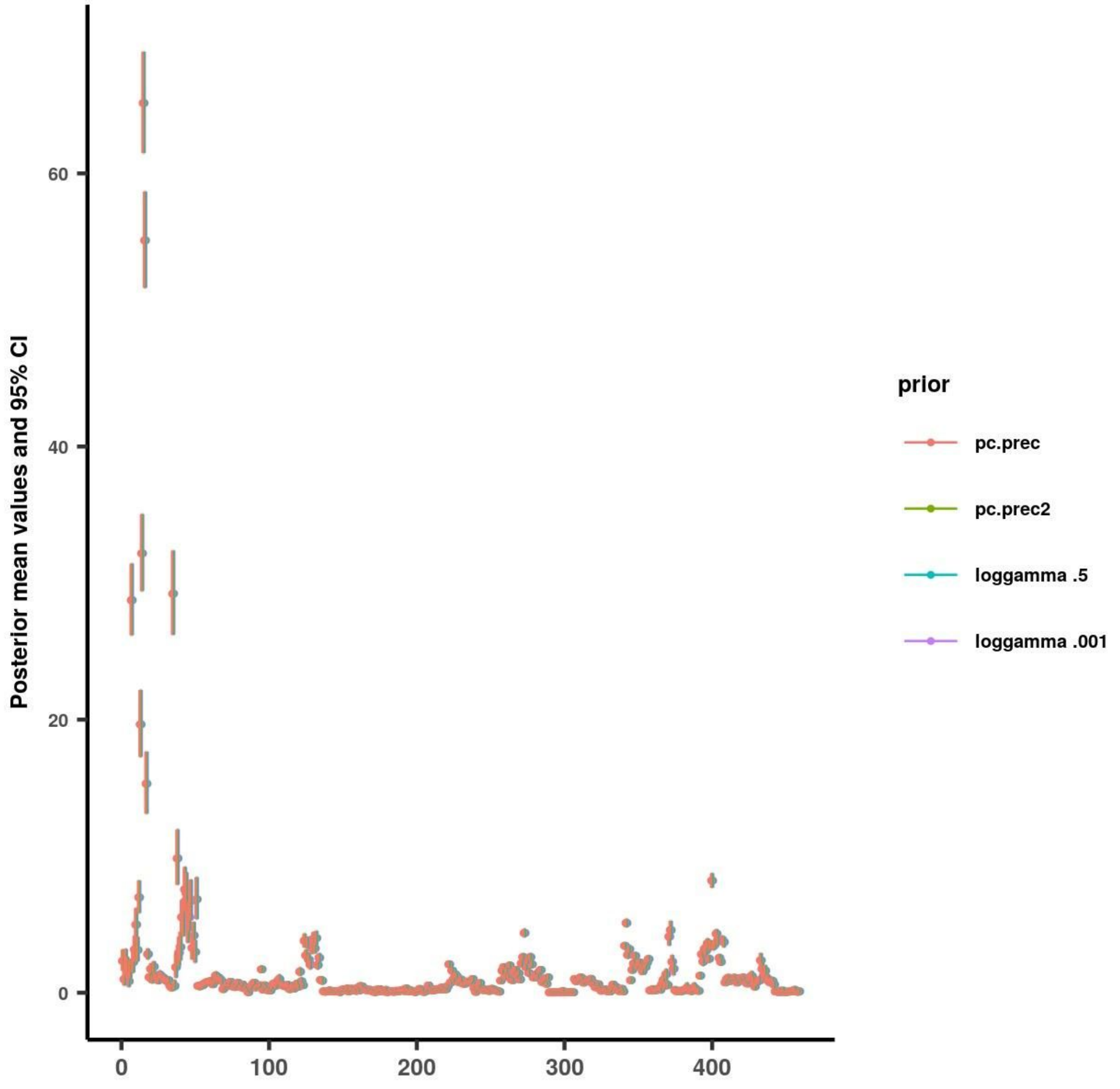

Fig. 5. Posterior mean fitted values and credible intervals (CI) of leptospirosis morbidity, conditioned by the prior specification, Brazil. 2000–2016.

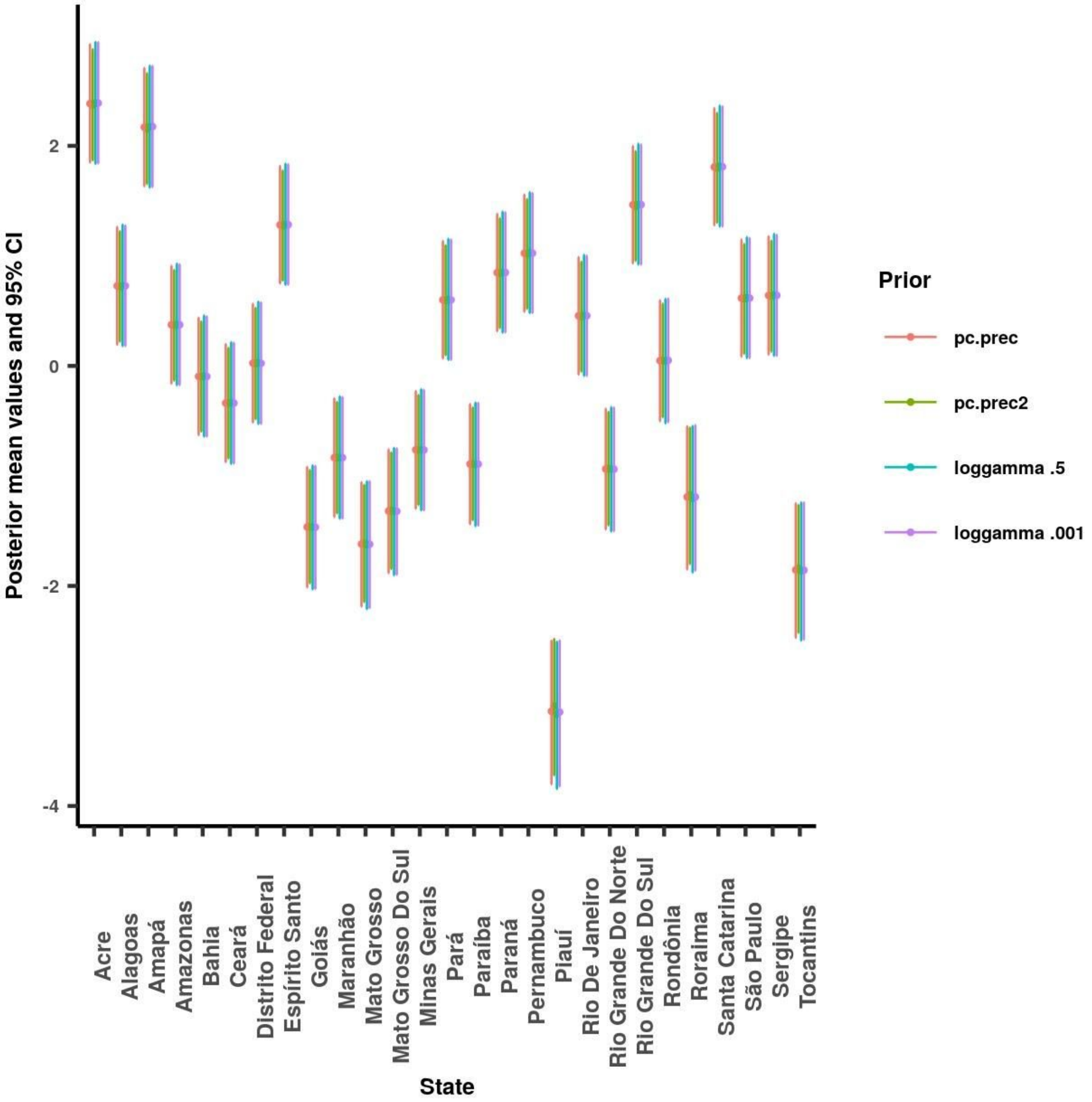

Fig. 6. Posterior mean spatial random effects and credible intervals (CI) of leptospirosis morbidity, conditioned by the prior specification, Brazil. 2000–2016.

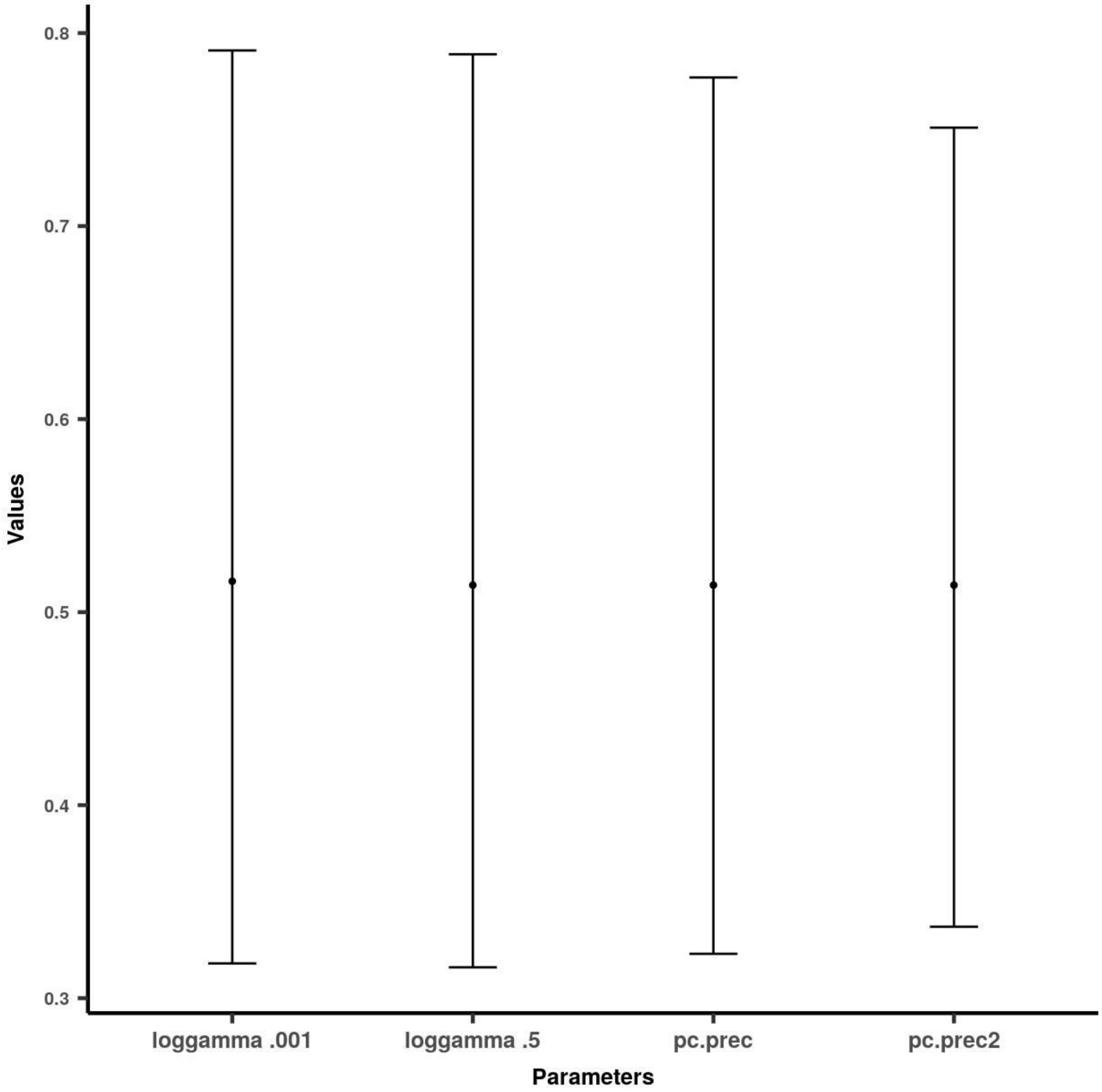

Fig. 7. Posterior mean fixed effects and credible intervals (CI) of leptospirosis morbidity, conditioned by the prior specification, Brazil. 2000–2016.

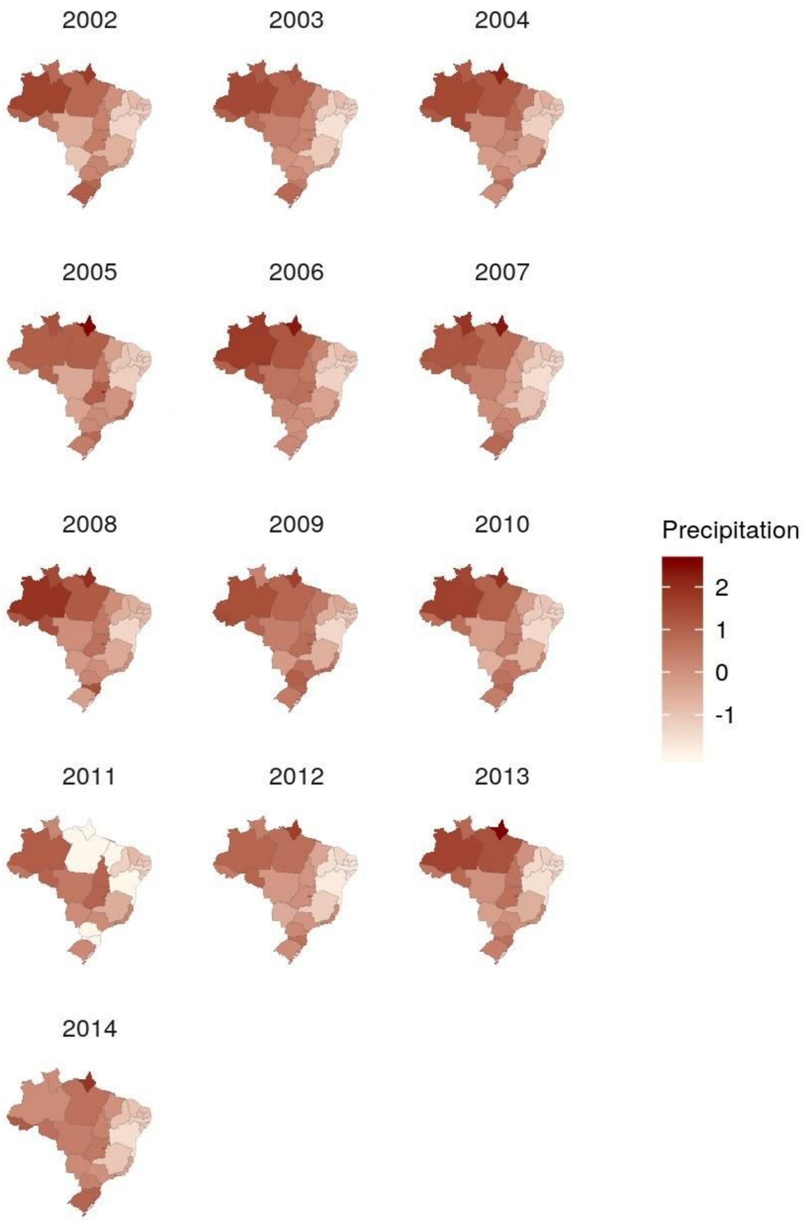

Fig. 8. Scaled precipitation, Brazil. 2002–2014.

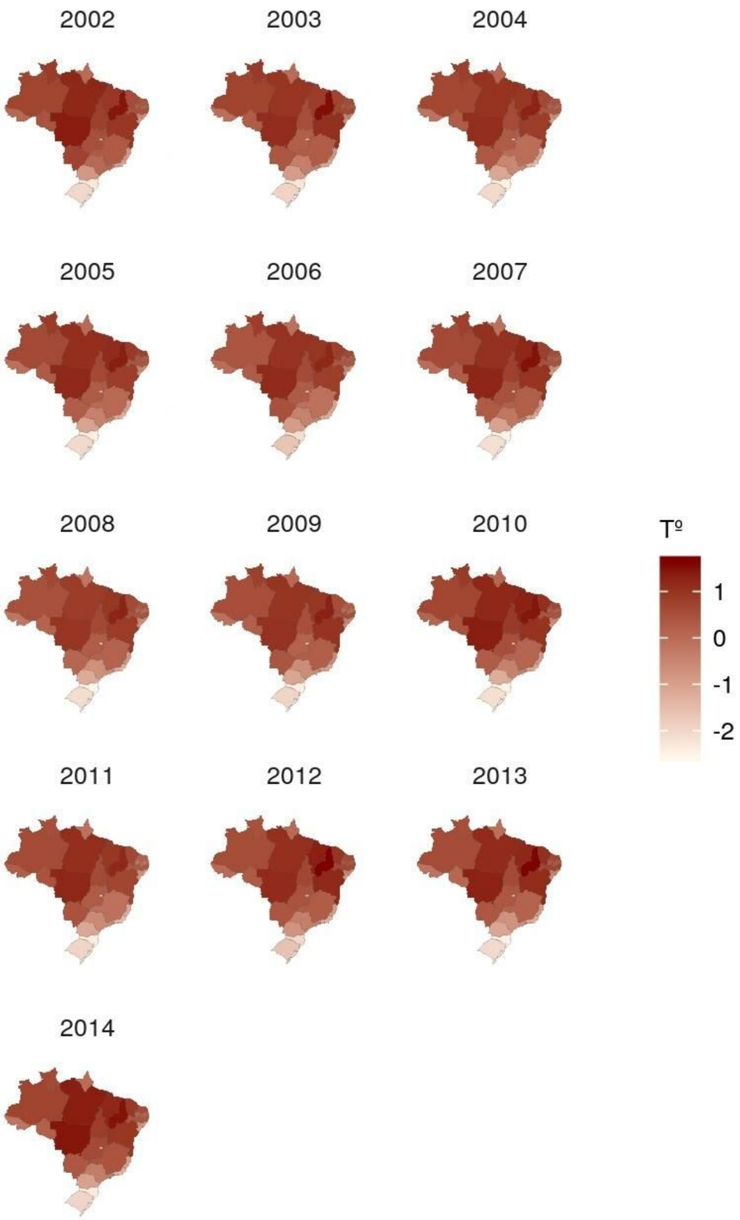

Fig. 9. Scaled temperature, Brazil. 2002–2014.

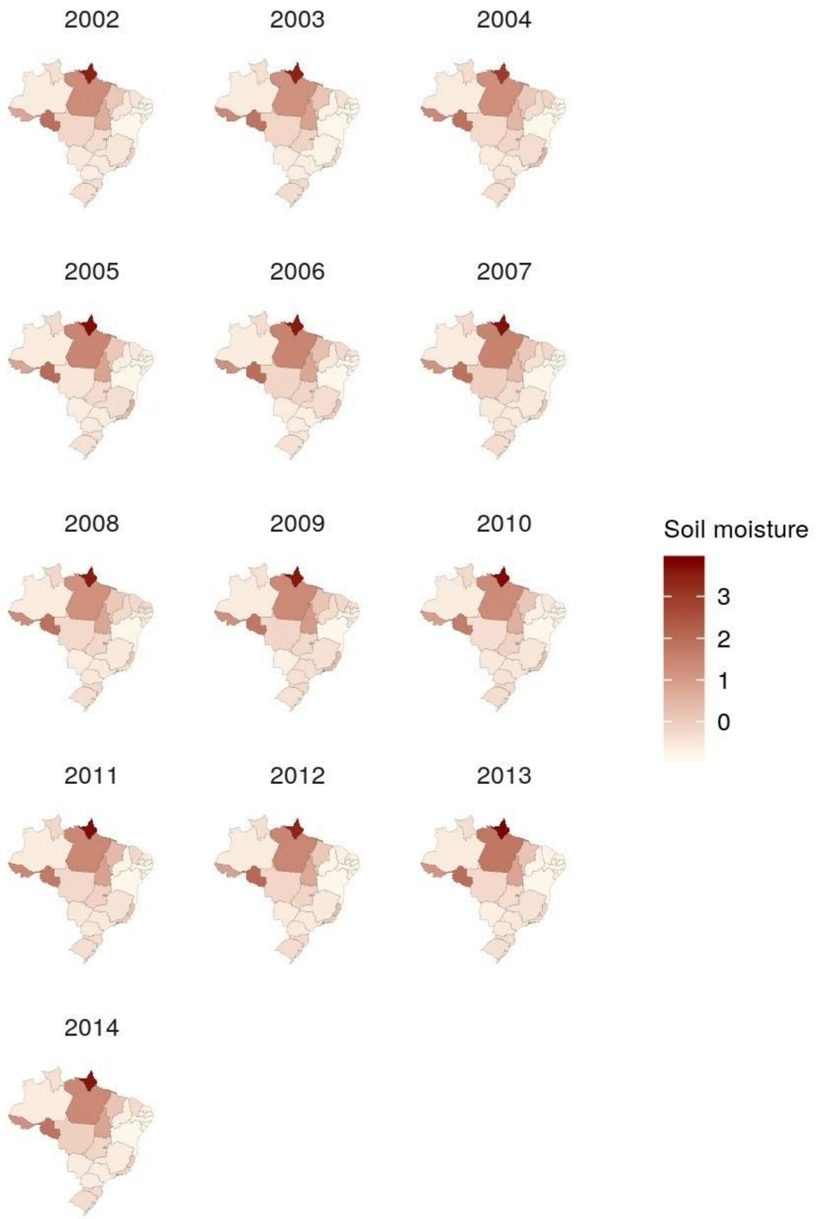

Fig. 10. Scaled soil moisture, Brazil. 2002–2014.

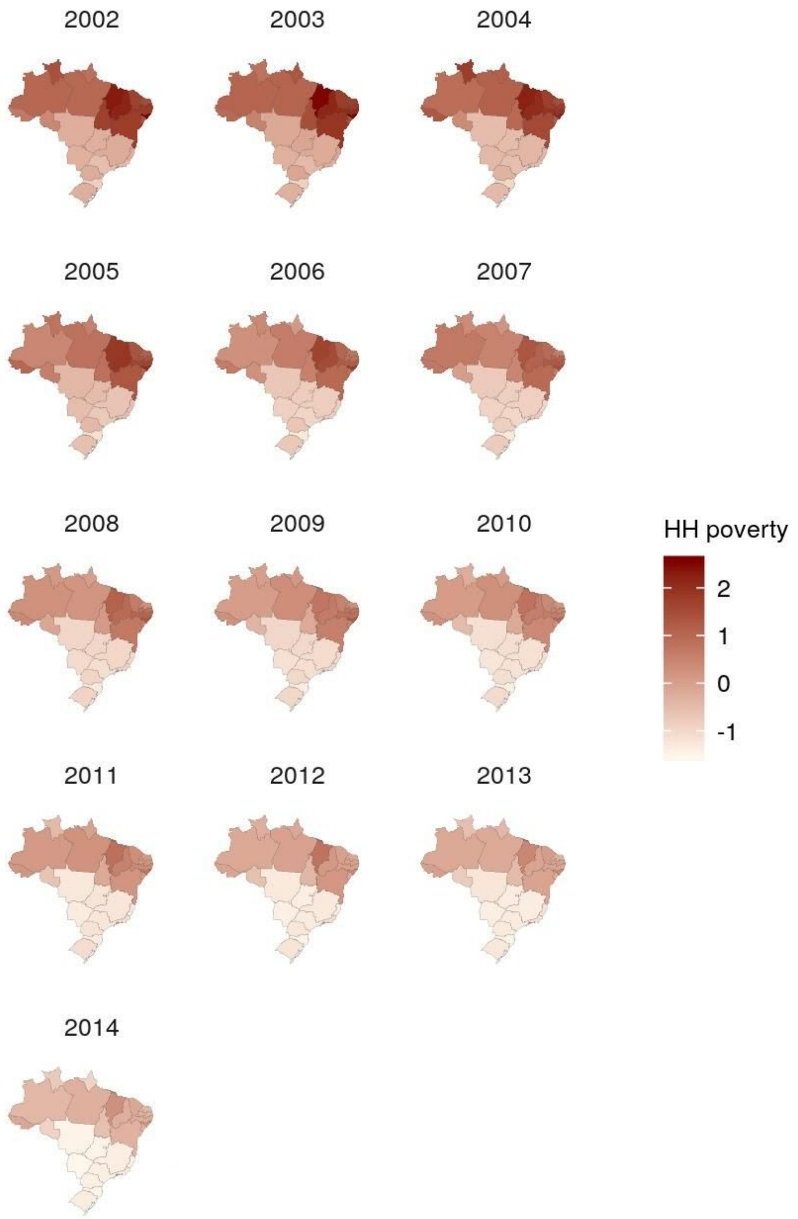

Fig. 11. Scaled proportion of households (HH) in poverty, Brazil. 2002–2014.

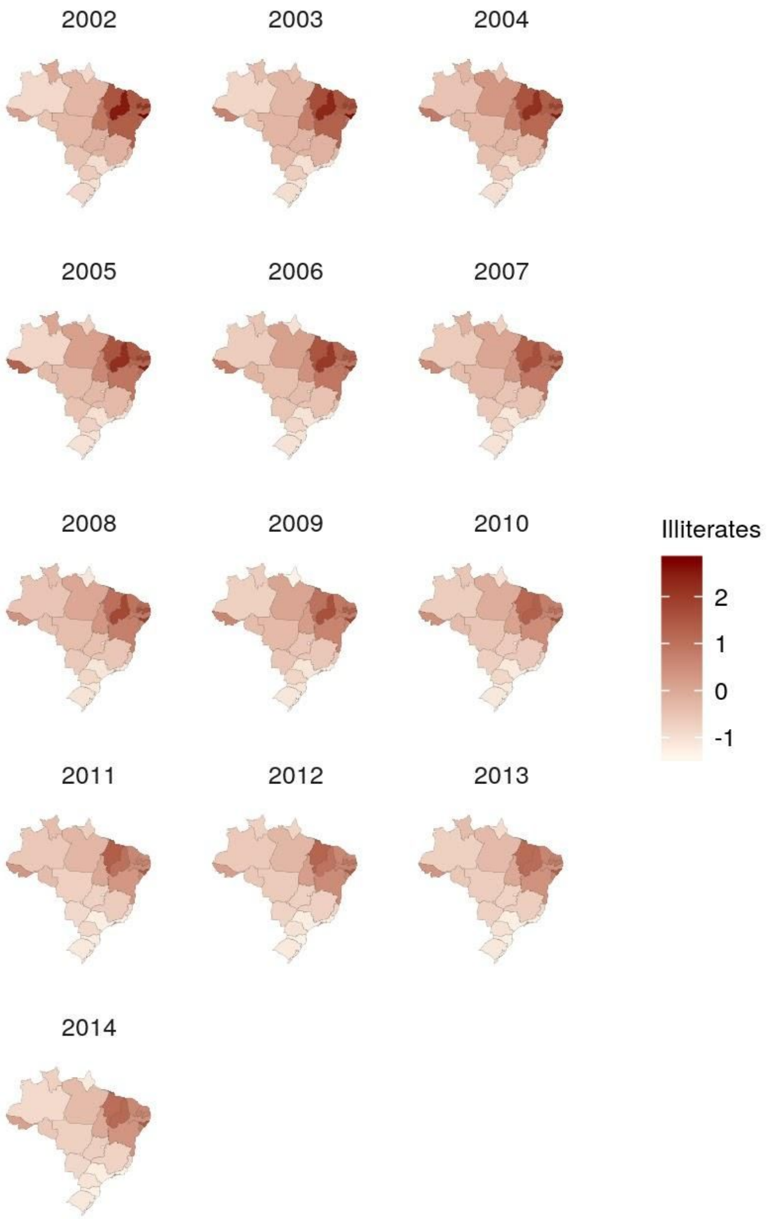

Fig. 12. Scaled proportion of illiterate residents. Brazil, 2002–2014.

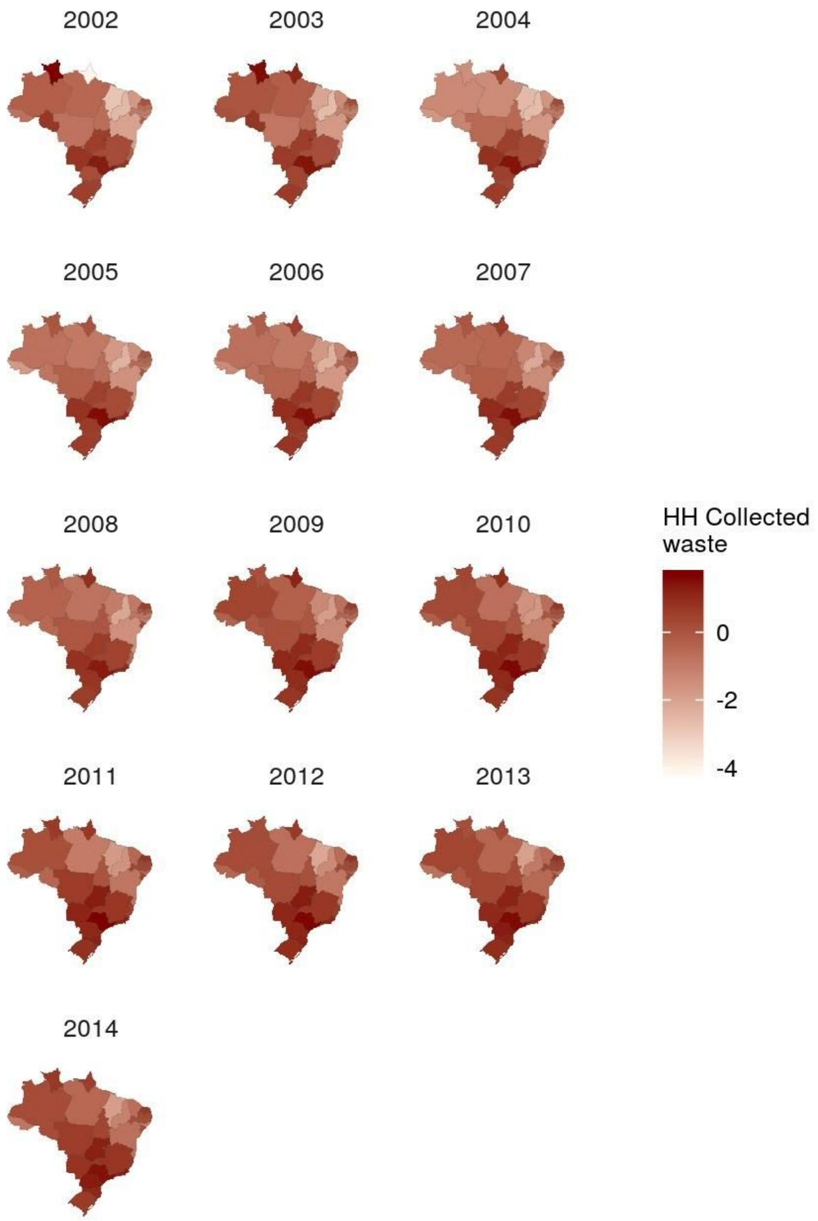

Fig. 13. Scaled proportion of households (HH) in which waste is directly collected, Brazil. 2002–2014.

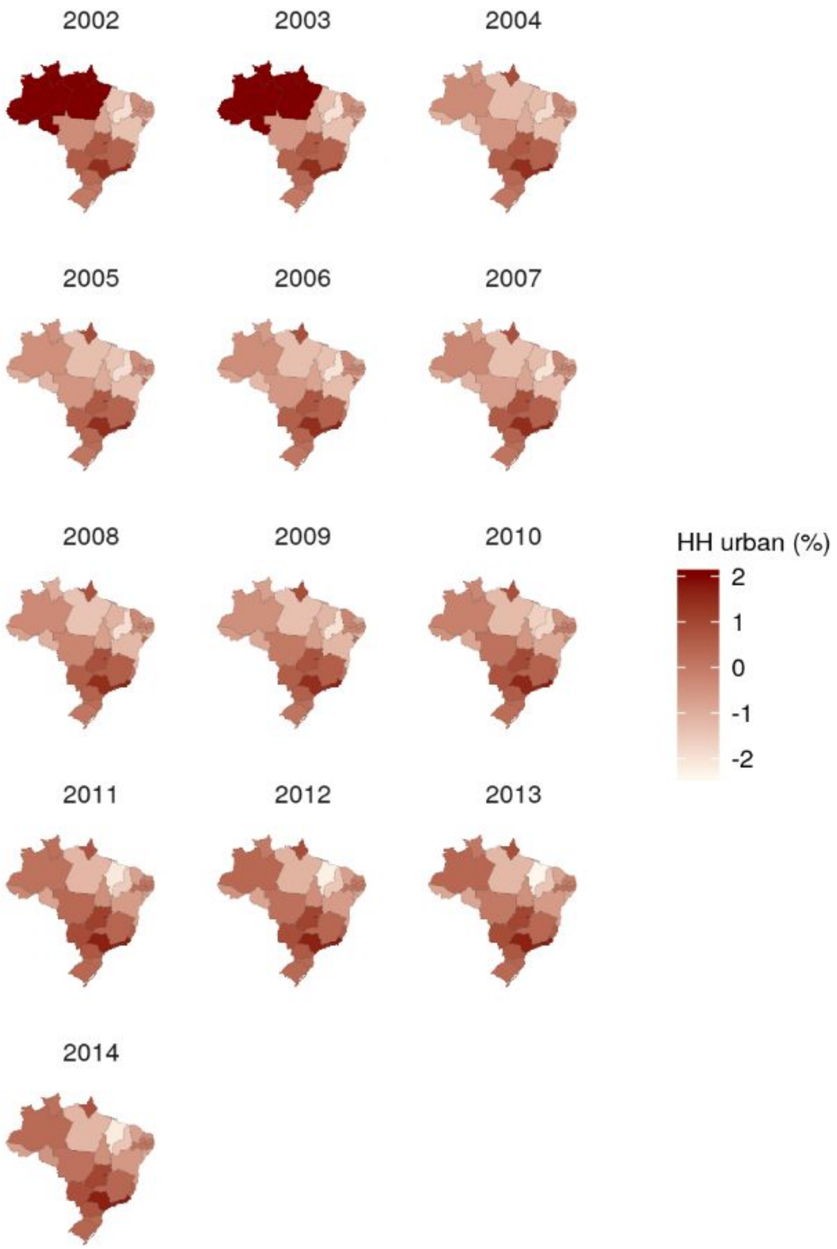

Fig. 14. Scaled proportion of urban households, Brazil. 2002–2014.

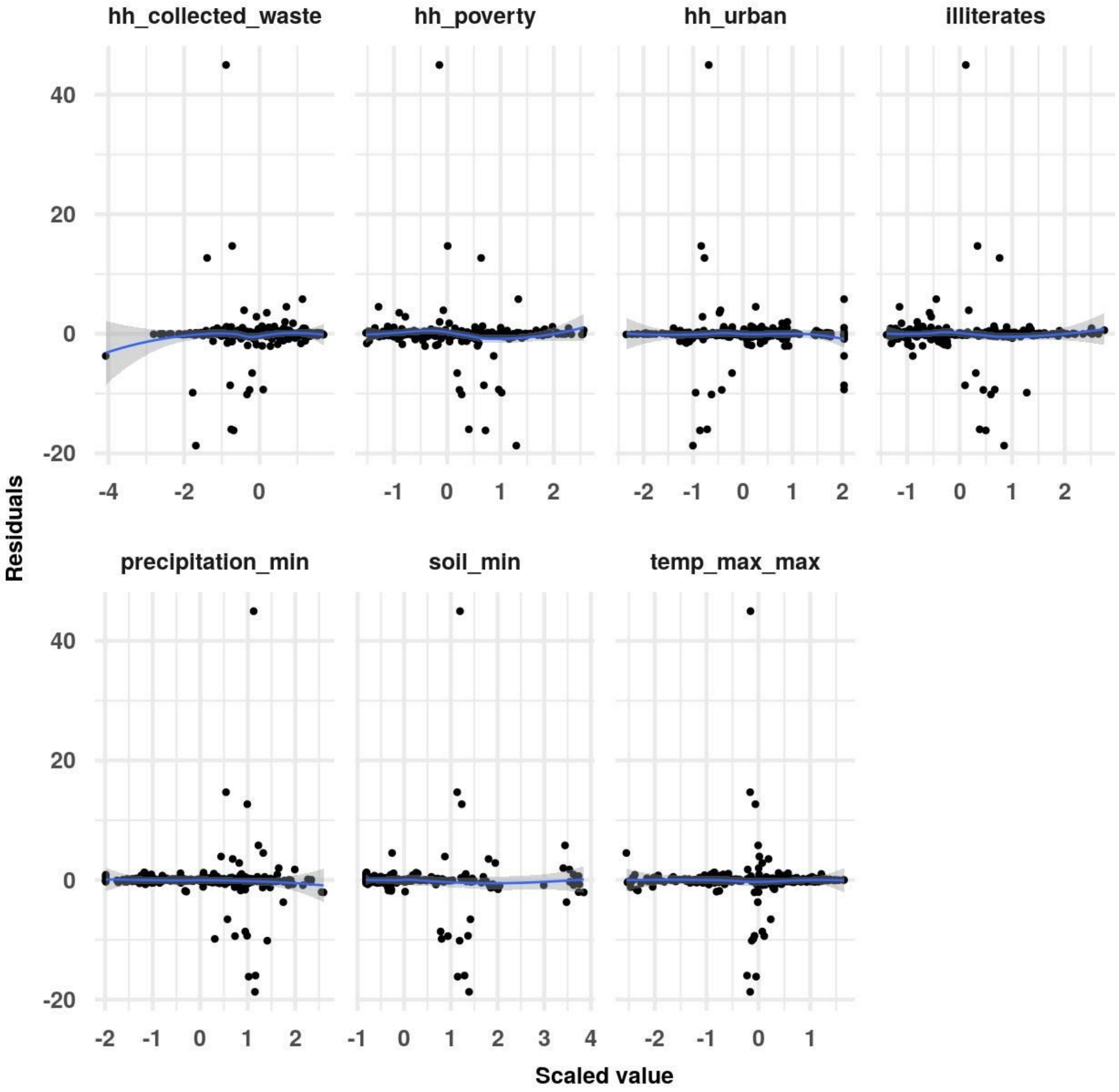

Fig. 15. Scatterplot of residuals against scaled covariates. Brazil, 2002–2014.

Table 1. Standardized morbidity ratio (SIR) and lethality of human leptospirosis in Brazilian Federative Units.

**Annual country average**	**2000**	**2001**	**2002**	**2003**	**2004**	**2005**	**2006**	**2007**	**2008**	**2009**	**2010**	**2011**	**2012**	**2013**	**2014**	**2015**	**2016**
**SIR (100.000)**	1.93	0.79	0.86	1.12	0.96	0.96	1.99	1.07	1.31	1.28	1.10	1.29	1.76	2.10	3.34	2.85	1.55
**Lethality %**	9.93	12.94	11.73	10.80	9.74	9.99	10.68	10.76	10.65	7.67	9.15	9.75	10.24	8.05	12.42	9.79	10.58
**Annual SIR (100.000) per state**	**2000**	**2001**	**2002**	**2003**	**2004**	**2005**	**2006**	**2007**	**2008**	**2009**	**2010**	**2011**	**2012**	**2013**	**2014**	**2015**	**2016**
Acre	2.17	0.65	1.93	1.47	0.37	1.40	28.96	2.02	3.01	4.92	2.99	6.98	19.84	32.40	65.44	55.39	15.47
Alagoas	2.86	1.14	1.77	0.95	1.93	1.21	1.09	0.89	1.33	1.20	1.11	1.04	0.90	0.83	0.90	0.48	0.32
Amapá	29.61	0.00	1.59	10.01	2.78	3.24	5.53	5.45	7.62	7.31	4.73	5.54	6.75	3.18	4.15	2.89	6.88
Amazonas	0.49	0.45	0.53	0.52	0.64	0.71	0.77	0.81	0.77	0.86	0.60	0.83	1.26	0.93	1.10	0.98	0.84
Bahia	0.26	0.40	0.57	0.53	0.56	0.80	0.47	0.49	0.41	0.52	0.72	0.46	0.40	0.60	0.33	0.40	0.24
Ceará	0.01	0.32	0.43	0.63	0.74	0.39	0.53	0.47	0.55	1.73	0.21	0.55	0.40	0.18	0.24	0.15	0.38
Distrito Federal	0.67	0.62	0.53	0.89	1.03	0.63	0.59	0.49	0.45	0.53	0.56	0.23	0.37	0.48	0.27	0.44	0.65
Espírito Santo	0.40	1.53	0.79	0.49	3.83	2.74	3.68	2.42	2.05	3.28	3.96	3.26	4.02	2.02	2.56	0.91	0.83
Goiás	0.10	0.06	0.07	0.08	0.17	0.10	0.11	0.07	0.09	0.10	0.11	0.03	0.21	0.24	0.16	0.19	0.32
Maranhão	0.08	0.27	0.29	0.20	0.24	0.12	0.36	0.15	0.50	0.47	0.32	0.28	0.19	0.15	0.21	0.13	0.13
Mato Grosso	0.05	0.02	0.27	0.07	0.30	0.20	0.19	0.06	0.23	0.02	0.05	0.10	0.10	0.05	0.05	0.14	0.11
Mato Grosso Do Sul	0.12	0.18	0.15	0.24	0.11	0.35	0.19	0.05	0.16	0.08	0.04	0.00	0.10	0.12	0.30	0.23	0.21
Minas Gerais	0.05	0.11	0.15	0.53	0.27	0.25	0.15	0.23	0.21	0.27	0.24	0.23	0.38	0.36	0.26	0.26	0.49
Pará	2.09	0.75	1.63	0.98	1.35	1.23	0.79	0.87	0.96	0.68	0.64	0.67	0.81	0.77	0.80	0.77	1.00
Paraíba	0.34	0.03	0.32	0.35	0.74	0.25	0.19	0.23	0.20	0.14	0.11	0.26	0.29	0.24	0.16	0.13	0.09
Paraná	0.05	0.89	1.62	1.88	1.18	1.70	1.18	2.00	0.96	0.91	1.56	1.67	1.30	1.42	0.96	2.14	2.62
Pernambuco	4.39	1.88	2.39	1.45	2.61	2.08	1.12	1.33	1.13	1.15	1.55	1.67	0.77	0.90	1.03	0.64	1.11
Piauí	0.03	0.00	0.00	0.00	0.00	0.02	0.03	0.00	0.02	0.17	0.00	0.03	0.04	0.03	0.01	0.03	0.02

Rio De Janeiro	1.01	0.85	0.90	0.97	1.13	1.05	0.74	0.88	0.85	0.94	0.89	1.02	0.69	0.70	0.40	0.39	0.61
Rio Grande Do Norte	0.33	0.13	0.22	0.20	0.24	0.09	0.13	0.05	0.30	0.63	0.26	0.43	0.24	0.10	0.18	0.29	0.04
Rio Grande Do Sul	3.44	5.11	2.77	3.19	0.90	1.67	2.14	2.70	1.94	2.07	2.22	1.96	1.56	1.93	1.89	2.21	2.46
Rondônia	0.09	0.07	0.18	0.04	0.08	0.14	0.27	0.11	0.60	0.95	0.49	1.40	0.53	4.23	4.70	2.34	1.85
Roraima	0.25	0.00	0.00	0.00	0.00	0.00	0.21	0.28	0.51	0.12	0.23	0.00	0.39	0.51	0.35	0.09	0.00
São Paulo	0.75	1.00	1.10	0.85	1.06	1.01	1.10	1.04	0.75	1.01	1.07	0.92	1.12	1.06	0.74	0.67	0.89
Santa Catarina	1.23	2.82	2.27	3.21	3.09	3.62	2.47	3.48	8.22	3.42	3.57	4.32	3.86	2.53	2.25	3.84	3.71
Sergipe	1.18	1.23	0.79	0.38	0.76	0.82	0.87	2.42	1.76	1.27	1.63	0.94	0.97	0.75	0.82	0.84	0.54
Tocantins	0.10	0.08	0.00	0.19	0.00	0.20	0.03	0.00	0.04	0.04	0.07	0.08	0.17	0.27	0.09	0.03	0.09
**Lethality (%) per state**	**2000**	**2001**	**2002**	**2003**	**2004**	**2005**	**2006**	**2007**	**2008**	**2009**	**2010**	**2011**	**2012**	**2013**	**2014**	**2015**	**2016**
Acre	0.00	0.00	0.00	6.25	0.00	10.53	0.64	0.00	10.00	8.57	11.36	4.51	2.00	0.59	0.58	0.21	0.56
Alagoas	4.48	9.86	9.76	14.58	3.03	12.68	7.59	12.00	6.25	6.41	4.35	8.33	16.67	14.29	8.57	5.71	12.50
Amapá	0.85	0.00	0.00	4.35	0.00	5.26	2.47	0.00	1.11	1.06	0.00	3.09	2.53	0.00	8.22	0.00	3.95
Amazonas	11.43	6.90	3.85	7.14	8.57	8.89	6.56	10.42	16.00	18.33	14.29	8.00	9.21	11.11	5.00	7.23	12.50
Bahia	11.63	11.30	12.40	17.36	18.32	16.43	14.19	15.87	15.79	16.77	14.50	12.57	11.58	12.57	11.86	16.28	15.38
Ceará	0.00	15.09	9.43	16.67	13.73	14.52	11.54	7.04	4.49	4.32	19.44	5.88	5.17	6.25	12.00	6.67	22.45
Distrito Federal	2.86	13.79	15.79	2.94	12.20	6.90	5.88	4.35	4.35	10.34	3.45	31.25	5.88	14.29	26.32	21.43	7.14
Espírito Santo	12.50	9.52	9.76	10.71	6.82	5.00	3.00	4.05	5.88	2.58	1.09	6.12	6.28	5.10	3.46	9.09	2.13
Goiás	7.14	25.00	28.57	0.00	23.53	16.67	6.25	25.00	18.18	0.00	0.00	0.00	13.64	15.63	16.00	3.57	9.68
Maranhão	16.67	14.71	14.29	23.81	15.38	20.00	13.21	22.22	22.95	8.06	2.38	20.83	18.18	0.00	11.43	25.00	28.57
Mato Grosso	0.00	0.00	8.33	50.00	26.67	8.33	21.43	0.00	0.00	0.00	0.00	22.22	16.67	0.00	40.00	27.27	50.00
Mato Grosso Do Sul	14.29	33.33	0.00	10.00	0.00	0.00	0.00	0.00	0.00	0.00	33.33	0.00	0.00	0.00	10.53	0.00	0.00
Minas Gerais	17.39	20.45	11.11	16.57	7.95	20.00	15.49	12.50	20.00	11.93	12.77	11.40	11.90	12.08	10.40	5.98	13.99

Pará	7.14	10.68	4.17	16.22	15.72	24.85	13.53	16.96	8.89	10.58	16.67	11.45	7.62	12.10	9.33	19.26	8.62
Paraíba	6.67	0.00	5.26	13.64	13.04	5.56	23.53	25.00	33.33	16.67	11.11	19.23	31.58	15.00	12.50	8.33	0.00
Paraná	25.00	15.59	13.89	10.73	14.15	10.45	12.41	7.77	9.23	9.55	19.06	12.98	10.53	14.29	10.48	9.82	8.76
Pernambuco	12.76	11.08	11.69	17.33	17.20	12.20	15.11	13.11	6.88	8.21	7.06	10.90	14.78	8.38	10.76	15.63	10.20
Piauí	33.33	0.00	0.00	0.00	0.00	0.00	0.00	0.00	0.00	0.00	0.00	0.00	0.00	0.00	50.00	0.00	0.00
Rio De Janeiro	14.96	24.72	24.76	19.92	21.77	18.65	19.56	18.55	21.32	15.69	20.71	7.93	16.04	18.26	25.81	16.06	16.08
Rio Grande Do Norte	4.17	44.44	9.09	9.09	0.00	0.00	10.00	50.00	10.53	2.44	11.76	11.11	14.29	0.00	0.00	4.55	33.33
Rio Grande Do Sul	5.41	6.61	4.59	5.08	9.04	8.62	5.81	6.36	7.44	4.35	5.36	4.69	6.09	5.75	5.31	6.24	4.12
Rondônia	25.00	33.33	80.00	0.00	0.00	0.00	0.00	0.00	11.11	10.00	6.25	3.51	13.33	2.70	2.09	3.37	2.13
Roraima	0.00	0.00	0.00	0.00	0.00	0.00	33.33	0.00	20.00	0.00	0.00	0.00	0.00	0.00	0.00	0.00	0.00
São Paulo	12.03	13.70	16.39	13.93	11.23	10.12	12.16	13.18	13.70	11.06	11.07	11.49	8.91	10.90	11.76	13.52	10.39
Santa Catarina	7.32	9.06	7.00	5.54	5.57	5.15	3.75	2.96	2.63	3.99	4.79	2.60	2.20	2.95	2.85	2.69	2.23
Sergipe	15.09	20.41	16.67	0.00	19.23	12.50	30.95	23.26	17.65	26.42	16.42	33.33	31.43	35.29	30.23	36.59	11.11
Tocantins	0.00	0.00	0.00	0.00	0.00	16.67	0.00	0.00	0.00	0.00	0.00	0.00	0.00	0.00	0.00	0.00	0.00

Table 2. Priority index for morbidity (standardized morbidity ratio) and lethality of human leptospirosis in Brazilian Federative Units.

**Morbidity** **Priority Index (PI) per state**	**2000**	**2001**	**2002**	**2003**	**2004**	**2005**	**2006**	**2007**	**2008**	**2009**	**2010**	**2011**	**2012**	**2013**	**2014**	**2015**	**2016**
Acre	7.9	8.4	79.3	17.8	5.2	45.3	100	41.2	38.5	68.5	65.7	100	100	100	100	100	100
Alagoas	9.7	18.8	63.1	3.5	50.1	31.4	3	3.6	16	15.7	19.1	9.9	1.2	0.2	0.3	0	0
Amapá	100	0.1	66.8	100	77.2	92.5	19.2	100	92.2	100	100	79.4	34.2	10.2	6.4	5.4	44.7
Amazonas	0	0	0	0	0	0.1	0	0.8	0.3	1.2	0	0.5	6.2	0.7	1.4	0.7	0.6
Bahia	0	0	0	0	0	0	0	0	0	0	0	0	0	0	0	0	0
Ceará	0	0	0	0	0	0	0	0	0	23.5	0	0	0	0	0	0	0
Distrito Federal	0	0	0	1.5	12.4	0	0	0	0	0	0	0	0	0	0	0	0
Espírito Santo	0	30.1	2.7	0	100	75.5	12.7	44.4	25.1	44.9	82.9	46.6	20.4	6.3	3.9	0.4	0.9
Goiás	0	0	0	0	0	0	0	0	0	0	0	0	0	0	0	0	0
Maranhão	0	0	0	0	0	0	0	0	0	0	0	0	0	0	0	0	0
Mato Grosso	0	0	0	0	0	0	0	0	0	0	0	0	0	0	0	0	0
Mato Grosso Do Sul	0	0	0	0	0	0	0	0	0	0	0	0	0	0	0	0	0
Minas Gerais	0	0	0	0	0	0	0	0	0	0	0	0	0	0	0	0	0
Pará	7.1	0	58.6	4.3	35.4	33.6	0	1	3.8	0	0	0	0.1	0	0	0	3.2
Paraíba	0	0	0	0	0.1	0	0	0	0	0	0	0	0	0	0	0	0
Paraná	0	0.9	58.3	19	30.7	46.8	4.1	36.6	3.7	1.3	32.7	24	6.6	4.4	0.4	3.9	17.1
Pernambuco	15	36.8	86.3	14.8	68.4	57.3	3.8	24.5	13.2	15.6	32.7	23.9	0	0.3	1.1	0	6.7
Piauí	0	0	0	0	0	0	0	0	0	0	0	0	0	0	0	0	0
Rio De Janeiro	1.9	0	1.5	2.9	29	23.1	0	0.4	0	1.7	0.4	9.1	0	0	0	0	0
Rio Grande Do Norte	0	0	0	0	0	0	0	0	0	0	0	0	0	0	0	0	0

Rio Grande Do Sul	11.8	100	100	32.4	2.8	46.3	7.4	49.6	23.7	28.5	46.6	28.1	8	6	2.9	4	16.1
Rondônia	0	0	0	0	0	0	0	0	0.1	4	0	19.2	0	12.8	7	4.1	11.5
Roraima	0	0	0	0	0	0	0	0	0	0	0	0	0	0	0	0	0
São Paulo	4.2	55.2	82.4	32.7	81.2	100	8.6	63.9	100	47.1	75	61.8	19.7	7.9	3.5	7	24.2
Santa Catarina	0	9.5	39.2	0	26.1	15.5	3.8	16.4	0	8.6	22	0	5.7	3.2	0	0	0
Sergipe	3.4	21.3	3.4	0	1.7	3.2	0.6	43.5	21.2	16.6	33.8	4.8	2.3	0.1	0.1	0.2	0
Tocantins	0	0	0	0	0	0	0	0	0	0	0	0	0	0	0	0	0

**Lethality Priority Index (PI) per state**	**2000**	**2001**	**2002**	**2003**	**2004**	**2005**	**2006**	**2007**	**2008**	**2009**	**2010**	**2011**	**2012**	**2013**	**2014**	**2015**	**2016**
Acre	0	0	0	0	0	0	0	0	0	0	0	0	0	0	0	0	0
Alagoas	6.3	16.8	20	26.1	19.3	29.8	23.3	24	19.9	15	20	24.3	31	30.8	27.3	25.3	26.5
Amapá	0	0	0	0	0	0	0	0	0	0	0	0	0	0	0	0	0
Amazonas	31.3	26	21.7	27.1	30.8	37.3	36.1	45.1	54.4	53.1	48.6	34.3	28.8	24.5	17.9	20	23.7
Bahia	70.7	57.6	59.6	74.5	78.9	82.4	69.3	70.2	72.2	67.2	61.7	48.4	43.7	42.1	43.8	48.2	53.7
Ceará	70.2	59.2	55.4	64	60.9	59.6	42.6	24.8	11.1	3.3	15.5	11.4	14.7	21.1	29.8	35.9	47.6
Distrito Federal	23.5	26.5	24.2	24.9	27.6	26.9	21.5	23.2	28.6	31.7	39.8	42	41.7	44.2	48.5	49	49.5
Espírito Santo	31.4	26.6	16.7	11.4	3	0.2	0	0	0	0	0	0	0.1	0.1	0	0.5	0.7
Goiás	76.6	65.1	62.2	66.5	67.6	68.3	55.2	53.4	50.6	39.9	39.2	33.9	34.7	34.6	33.2	29.7	30.6
Maranhão	92.3	73.3	71	80.8	79.5	84.2	71.8	73.1	73.7	58.3	55.3	52.2	48.8	45.3	49.3	56	65
Mato Grosso	78	65.8	65.3	77.3	78.5	79.9	68.7	65.1	65.2	63.6	68.4	63.2	63.6	65.8	73.8	79.9	92.8
Mato Grosso Do Sul	24.3	20.9	14.3	12.7	9.1	6.8	4.4	4.1	4	3.3	4.1	3	2.7	2.5	2.6	2.5	2.6
Minas Gerais	95.4	74.7	66.5	72.8	67.6	80.7	68.8	66.2	69.7	58.3	54.8	43.9	40	37.1	34.6	32.5	41.4
Pará	26.8	34.7	34.6	61.1	74	95	70.6	65.4	56.7	52.4	54.7	42.4	36.5	37.1	38.3	44.5	42.6
Paraíba	47.4	43.3	44.6	57.1	63.2	73.4	73.8	80.7	87.4	79.6	78.3	66.8	62.7	55.3	52	49.3	51.2
Paraná	87.8	66.5	58.7	57.4	59.1	57.2	49.8	40.8	46.1	50.2	65.1	48.2	41.3	41.1	36	32.5	30.7
Pernambuco	74.1	54.6	55.8	72.8	75	69	60.7	53.1	40.4	30.8	32.8	37.1	37.8	33.6	36.5	40.3	41.1
Piauí	10	8.2	6.8	7.2	6.7	6.6	5.2	5	4.9	4.1	4.7	4	4	4.1	4.5	4.6	4.8
Rio De Janeiro	100	100	100	100	100	99.1	87.6	85.7	90	74	72.9	43.7	51.3	58.1	67.6	59.3	62.5

Rio Grande Do Norte	44.4	41.9	34.8	35.1	31.4	32.5	28.4	29.3	27.3	19.5	23.4	19.3	16.6	13.3	11.9	12.9	14.3
Rio Grande Do Sul	0.1	0.4	0.1	0.2	7.1	11.5	1.4	1.5	1.8	0	0	0	0.2	0.1	0.1	0.1	0
Rondônia	77.6	62.8	56.7	50.4	39.6	32	19.8	15.1	11.8	5.6	2.8	0.5	0.1	0	0	0	0
Roraima	8.1	7.3	6.6	7.6	7.8	8.5	7.4	7.1	7.2	5.6	5.6	4.2	3.7	3.4	3.3	3.5	3.6
São Paulo	72.9	61.5	66.9	65.1	55.3	55.2	54.5	58.2	60	49.9	47.5	40.3	33	35.1	38.5	41.4	40.4
Santa Catarina	17.4	19.7	5.1	0.9	0.2	0	0	0	0	0	0	0	0	0	0	0	0
Sergipe	98.8	80.4	75.8	82.6	88.4	100	100	100	100	100	100	100	100	100	100	100	100
Tocantins	0.8	0.7	0.6	0.7	0.7	0.8	0.6	0.5	0.5	0.4	0.4	0.3	0.3	0.3	0.3	0.3	0.3

Table 3. Deviance information criterion (DIC) and the posterior predictive p-value, conditioned by the prior specification, Brazil. 2000–2016.

Prior	DIC	p-value
pc.prec	3,403·36	(7·2, 0·2)
pc.prec2	3,405·29	(7·4, 0·2)
loggamma .5	3,401·44	(6·8, 0·0)
loggamma .001	3,404·44	(7·4, 0·0)

Other appendices accompanying this manuscript

Animation 1. Posterior mean excess risk of leptospirosis morbidity, Brazil. 2000–2016. Pr (RR>1) representing the ER: Excess Risk, RR: Relative Risk, IID: Independent and Identically Distributed, RW1: Random Walk of first order.

Animation 2. Posterior mean relative risk of leptospirosis morbidity, Brazil. 2000–2016. RR: Relative Risk, IID: Independent and Identically Distributed, RW1: Random Walk of first order.

Animation 3. Posterior mean excess risk of leptospirosis lethality, Brazil. 2000–2016. Pr (Lethality>intercept) representing the ER: Excess Risk, IID: Independent and Identically Distributed, RW1: Random Walk of first order.

Animation 4. Posterior mean leptospirosis lethality, Brazil. 2000–2016. IID: Independent and Identically Distributed, RW1: Random Walk of first order.
