# Supplementary material for: Spatiotemporal dynamics and risk factors for human Leptospirosis in Brazil
Source: Sci Rep. 2018 Oct 11;8:15170. doi: 10.1038/s41598-018-33381-3 (PMC6181921; doi:10.1038/s41598-018-33381-3)
Supplement: Supplementary file 6 — Supplementary-Analysis [file 41598_2018_33381_MOESM6_ESM.zip › 33381 Supplementary zip-file/data/covariates/dengue.pdf]

Casos de Dengue. Brasil, Grandes Regiões e Unidades Federadas, 1990 a 2016*

Região e UF	1990	1991	1992	1993	1994	1995	1996	1997	1998	1999	2000
Região Norte	0	2.194	0	0	18	3.221	2.695	22.174	27.018	7.128	24.686
Rondônia	...	...	0	...	0	0	0	55	23	699	3.193
Acre	...	...	0	...	0	0	0	0	0	0	1.295
Amazonas	...	...	0	...	0	0	0	0	13.894	186	6.343
Roraima	...	...	0	...	0	0	409	380	258	1.999	4.108
Pará	...	...	0	...	0	28	321	20.877	10.934	4.157	8.505
Amapá	...	...	0	...	0	0	0	0	26	4	0
Tocantins	...	2.194	0	...	18	3.193	1.965	862	1.883	83	1.242
Região Nordeste	15.950	8.020	0	788	49.828	59.192	125.779	190.746	227.566	37.533	70.091
Maranhão	...	...	0	...	0	1.776	6.312	6.102	12.171	1.523	3.613
Piauí	...	...	0	...	26	3.260	5.777	2.841	14.626	2.179	6.756
Ceará	15.656	6.703	0	7	47.221	1.991	2.099	6.590	13.389	377	1.323
Rio Grande do Norte	...	...	0	...	345	5.181	6.608	25.579	17.850	7.943	16.563
Paraíba	...	...	0	...	0	1.701	12.068	52.701	58.612	25	32
Pernambuco	...	...	0	...	0	9.982	22.722	32.627	52.633	14.322	24.785
Alagoas	294	1.317	0	781	344	794	2.596	7.666	9.078	1.088	915
Sergipe	...	...	0	...	0	0	3.162	11.187	27.311	9.097	6.786
Bahia	...	...	0	...	1.892	34.507	64.435	45.453	21.896	979	9.318
Região Sudeste	22.723	89.839	1.696	5.124	968	46.845	34.294	22.633	229.630	25.119	32.906
Minas Gerais	...	286	0	3.863	0	2.832	5.250	5.355	147.402	2.128	8.003
Espírito Santo	...	...	0	...	0	2.725	5.715	12.934	39.216	691	17.497
Rio de Janeiro	19.685	85.891	1.658	623	287	35.240	16.225	2.304	32.382	519	3.220
São Paulo**	3.038	3.662	38	638	681	6.048	7.104	2.040	10.630	21.781	4.186
Região Sul	0	0	0	0	0	3.116	5.213	721	2.949	51	1.242
Paraná	...	...	0	...	0	3.116	5.201	716	2.702	15	1.184
Santa Catarina	...	...	0	...	0	0	3	5	140	8	23
Rio Grande do Sul	...	...	0	...	0	0	9	0	107	28	35
Região Centro-Oeste	1.606	4.346	0	1.462	5.877	24.934	15.781	12.965	20.552	4.839	6.303
Mato Grosso do Sul	1.606	4.346	0	570	1.154	5.115	3.364	4.985	2.578	4.688	4.194
Mato Grosso	...	...	0	892	1.367	11.628	6.016	3.562	8.787	4	291
Goiás	...	...	0	...	3.343	8.191	6.316	3.709	6.412	147	1.618
Distrito Federal	...	...	0	...	13	0	85	709	2.775	0	200
**Brasil**	**40.279**	**104.399**	**1.696**	**7.374**	**56.691**	**137.308**	**183.762**	**249.239**	**507.715**	**74.670**	**135.228**

Fonte: SES/SINAN (SINAN: a partir de 1999)

OBS: Dos dados obtidos pelo Sinan foram tabulados todos os casos, exceto os descartados.

* Atualizado em 13/01/2017. Dados sujeitos a alteração.

2001	2002	2003	2004	2005	2006	2007	2008	2009	2010	2011	2012
51.309	19.930	28.285	18.492	24.813	19.754	36.552	48.946	55.611	98.632	119.398	42.158
1.668	1.472	2.971	3.048	5.672	4.123	3.118	5.765	18.767	20.294	3.219	3.292
2.050	888	954	4.422	2.196	258	518	2.129	19.085	35.162	18.865	2.393
19.249	2.188	3.971	902	998	578	1.938	8.755	1.591	7.524	61.986	5.167
3.782	1.206	4.971	558	2.328	954	876	5.016	3.073	7.590	1.451	1.874
16.564	11.362	9.617	5.521	7.988	6.436	13.920	15.982	7.498	15.568	19.240	16.246
3.193	845	3.932	2.564	2.338	1.713	3.500	1.177	1.568	3.242	2.803	1.569
4.803	1.969	1.869	1.477	3.293	5.692	12.682	10.122	4.029	9.252	11.834	11.617
149.582	266.767	150.208	21.782	74.621	68.037	124.869	207.808	125.296	176.854	195.365	222.913
6.235	8.360	5.836	1.580	6.537	4.931	13.354	5.734	2.251	5.778	11.777	5.325
10.284	8.793	9.525	850	4.217	4.666	9.662	2.424	4.006	7.137	10.060	12.255
33.986	19.586	33.182	3.849	26.942	28.306	34.353	54.661	7.883	21.246	63.206	54.831
37.431	21.761	20.766	2.605	4.730	8.134	12.994	35.600	2.654	7.846	23.171	28.778
14.947	18.618	12.780	1.340	5.772	3.047	10.732	8.442	951	6.667	12.602	8.771
13.233	99.652	15.165	2.275	5.389	8.262	22.380	20.137	2.882	34.590	22.101	31.799
1.980	7.438	6.080	4.455	2.608	3.010	10.566	13.740	3.978	47.358	8.909	28.123
3.490	5.133	4.720	421	649	1.118	1.485	29.645	1.824	803	3.927	4.558
27.996	77.426	42.154	4.407	17.777	6.563	9.343	37.425	98.867	45.429	39.612	48.473
159.461	355.587	64.482	21.219	20.935	124.495	206.151	325.403	113.259	478.003	361.350	251.738
31.955	38.082	14.213	13.602	10.929	28.713	28.287	52.088	55.146	214.552	40.343	29.456
7.618	24.726	28.115	3.420	2.662	9.263	6.790	25.357	38.146	26.081	40.336	11.961
61.401	248.493	5.470	1.152	1.184	26.150	56.202	235.353	7.964	29.824	165.787	181.169
58.487	44.286	16.684	3.045	6.160	60.369	114.872	12.605	12.003	207.546	114.884	29.152
1.674	7.926	11.108	186	1.140	1.347	27.772	2.166	1.641	42.008	35.978	4.772
1.581	7.197	10.991	152	1.050	1.223	27.200	1.924	1.525	38.173	35.438	4.508
38	293	58	14	49	54	149	104	49	189	177	94
55	436	59	20	41	70	423	138	67	3.646	363	170
23.757	46.262	20.892	8.495	25.530	45.047	101.579	48.357	110.462	216.051	51.941	68.010
9.334	12.182	2.091	311	617	11.358	69.412	829	14.027	63.519	8.510	9.202
2.362	8.978	9.408	2.294	6.524	9.451	16.227	7.052	52.219	35.443	6.146	32.856
10.530	21.936	8.504	5.635	18.058	23.770	14.820	39.075	43.411	102.071	33.960	24.517
1.531	3.166	889	255	331	468	1.120	1.401	805	15.018	3.325	1.435
**385.783**	**696.472**	**274.975**	**70.174**	**147.039**	**258.680**	**496.923**	**632.680**	**406.269**	**1.011.548**	**764.032**	**589.591**

2013	2014	2015	2016
49.547	48.376	31.411	39.011
8.732	1.985	2.211	7.769
2.568	28.269	5.317	2.335
17.832	6.661	3.792	8.125
945	1.123	1.097	246
9.166	4.496	7.803	10.852
1.708	2.190	3.297	1.798
8.596	3.652	7.894	7.886
152.357	89.935	327.212	324.815
3.588	2.652	7.943	24.167
4.987	7.657	7.646	5.242
30.219	22.756	63.116	50.897
18.905	11.498	22.700	57.508
13.466	5.625	23.426	35.688
7.985	10.488	110.899	65.152
11.296	13.186	27.130	17.301
801	2.246	9.141	3.509
61.110	13.827	55.211	65.351
918.226	311.639	1.047.279	858.273
416.252	58.177	192.779	528.441
67.995	18.879	35.441	41.736
213.058	7.717	73.437	85.200
220.921	226.866	745.622	202.896
66.903	22.988	51.681	72.650
66.100	22.701	45.542	64.305
358	134	4.441	5.150
445	153	1.698	3.195
265.456	116.169	231.105	205.786
78.958	3.423	32.241	44.814
35.190	7.160	21.583	19.940
139.357	93.929	167.427	123.195
11.951	11.657	9.854	17.837
**1.452.489**	**589.107**	**1.688.688**	**1.500.535**
